# Effectiveness of monovalent COVID-19 booster/additional vaccine doses in the United States

**DOI:** 10.1016/j.jvacx.2024.100447

**Published:** 2024-01-20

**Authors:** J. Bradley Layton, Lauren Peetluk, Hui Lee Wong, Yixin Jiao, Djeneba Audrey Djibo, Christine Bui, Patricia C. Lloyd, Joann F. Gruber, Michael Miller, Rachel P. Ogilvie, Jie Deng, Ron Parambi, Jennifer Song, Lisa B. Weatherby, An-Chi Lo, Kathryn Matuska, Michael Wernecke, Tainya C. Clarke, Sylvia Cho, Elizabeth J. Bell, John D. Seeger, Grace Wenya Yang, Dóra Illei, Richard A. Forshee, Steven A. Anderson, Cheryl N. McMahill-Walraven, Yoganand Chillarige, Kandace L. Amend, Mary S. Anthony, Azadeh Shoaibi

**Affiliations:** a RTI Health Solutions, Research Triangle Park, NC, USA; b Optum Epidemiology, Boston, MA, USA; c US Food and Drug Administration, Center for Biologics Evaluation and Research, Silver Spring, MD, USA; d Acumen, LLC, Burlingame, CA, USA; e Safety, Surveillance & Collaboration, CVS Health, Blue Bell, PA, USA; f Optum Serve, Falls Church VA, USA; g RTI International, Washington, DC, USA

**Keywords:** COVID-19 vaccines, Pharmacoepidemiology, Vaccine effectiveness

## Abstract

**Background:**

Monovalent booster/additional doses of COVID-19 vaccines were first authorized in August 2021 in the United States. We evaluated the real-world effectiveness of receipt of a monovalent booster/additional dose of COVID-19 vaccine compared with receiving a primary vaccine series without a booster/additional dose.

**Methods:**

Cohorts of individuals receiving a COVID-19 booster/additional dose after receipt of a complete primary vaccine series were identified in 2 administrative insurance claims databases (Optum, CVS Health) supplemented with state immunization information system data between August 2021 and March 2022. Individuals with a complete primary series but without a booster/additional dose were one-to-one matched to boosted individuals on calendar date, geography, and clinical factors. COVID-19 diagnoses were identified in any medical setting, or specifically in hospitals/emergency departments (EDs). Propensity score-weighted hazards ratios (HRs) and 95% confidence intervals (CI) were estimated with Cox proportional hazards models; vaccine effectiveness (VE) was estimated as 1 minus the HR by vaccine brand overall and within subgroups of variant-specific eras, immunocompromised status, and homologous/heterologous booster status.

**Results:**

Across both data sources, we identified 752,165 matched pairs for BNT162b2, 410,501 for mRNA-1273, and 11,398 for JNJ-7836735. For any medically diagnosed COVID-19, meta-analyzed VE estimates for BNT162b2, mRNA-1273, and JNJ-7836735, respectively, were: BNT162b2, 54% (95% CI, 53%-56%); mRNA-1273, 58% (95% CI, 56%-59%); JNJ-7836735, 34% (95% CI, 23%-44%). For hospital/ED-diagnosed COVID-19, VE estimates ranged from 70% to 76%. VE was generally lower during the Omicron era than the Delta era and for immunocompromised individuals. There was little difference observed by homologous or heterologous booster status.

**Conclusion:**

The original, monovalent booster/additional doses were reasonably effective in real-world use among the populations for which they were indicated during the study period. Additional studies may be informative in the future as new variants emerge and new vaccines become available.

Registration: The study protocol was publicly posted on the BEST Initiative website (https://bestinitiative.org/wp-content/uploads/2022/03/C19-VX-Effectiveness-Protocol_2022_508.pdf).

## Introduction

Multiple vaccines against coronavirus disease 2019 (COVID-19), caused by severe acute respiratory syndrome coronavirus 2 (SARS-CoV-2), have been authorized in the United States (US). In response to changes in circulating viral variants, observed waning of effectiveness of the primary series, and concerns about insufficient immune response among immunocompromised individuals, the US Food and Drug Administration (FDA) authorized and recommended monovalent booster or additional doses of the COVID-19 vaccines after receipt of the primary series. Vaccination recommendations and authorizations for booster/additional doses have varied over time by age group and risk status, with booster/additional doses first authorized for immunocompromised individuals, and subsequently for adults aged 65 years and older, adults aged 18–64 years, and children. Vaccination authorizations and recommendations have continued to evolve throughout the pandemic, as authorizations of updated vaccines have replaced the original mRNA monovalent booster doses [1], [2], [3]. With rapid changes in vaccination authorizations and recommendations, evaluations of the effectiveness of implemented vaccination strategies are informative for future vaccination efforts.

As part of its continued monitoring and surveillance of authorized vaccines, the US FDA and partners within the Biologics Effectiveness and Safety (BEST) Initiative undertook this study to evaluate the real-world effectiveness of the receipt of one of the initial monovalent COVID-19 booster/additional vaccine doses in the United States. The objective of this study was to estimate the effectiveness of receiving a monovalent booster/additional dose of a COVID-19 vaccine compared with not receiving a booster/additional dose, in individuals who had previously received a complete primary vaccine series (either 1- or 2-dose, depending on the primary series brand).

## Materials and methods

### Population and data source

This cohort study was conducted in 2 US administrative claims databases (Optum preadjudicated commercial insurance claims; CVS Health [Aetna] commercial health insurance claims) supplemented with vaccination records from Immunization Information Systems (IISs) in the US [4]. In both data sources, the study population was restricted to individuals residing in jurisdictions with IIS linkage (eTable 1). Optum claims were combined with data from 10 IIS jurisdictions in 10 US states between 11 December 2020 (the date COVID-19 vaccines were first available in the US) and 28 February 2022 (the latest date of updated claims and IIS data at the time of analysis); CVS Health claims were combined with IIS data from 11 jurisdictions in 9 US states between 11 December 2020, and 31 March 2022.

Each COVID-19 vaccine brand was evaluated in separate matched cohorts: BNT162b2, mRNA-1273, and JNJ-7836735 [1], [2], [3]. Depending on the brand, additional doses of an extended primary series were generally authorized first for immunocompromised individuals, with subsequent authorizations for booster doses for adults aged 65 years and older, those at high risk of severe COVID-19, and other age groups. We evaluated booster and additional doses together, as both additional and booster doses were identified based on the chronological ordering of vaccinations, such as a third dose after mRNA-based vaccination or 2nd dose after JNJ-7836735 vaccination. The study period for each brand began on the respective date of the initial authorization for a booster/additional dose (12 August 2021, for BNT162b2 and mRNA-1273; 20 October 2021, for JNJ-7836735; see eTable 2) [1], [2], [3]. Booster/additional dose recipients (“boosted” individuals) were identified at their first receipt of a booster/additional dose of a COVID-19 vaccine after 28 days (the minimum recommended spacing for an additional dose in immunocompromised individuals during the study period) or greater following completion of the primary series. The brand of the booster/additional dose was not required to match the brand of the primary series. The date of receipt of the booster/additional dose was defined as Time 0 for boosted individuals. Individuals who received a primary series but who had not received a booster/additional dose at Time 0 (“unboosted” comparators) were matched to boosted individuals using 1:1 exact matching with replacement based on calendar date of vaccination, age categories, sex, county and state of residence, brand of the primary series, and time since primary series completion (14-day increments), immunocompromised status, and presence of at least 1 of the conditions identified by the Centers for Disease Control and Prevention (CDC) as increasing individuals’ risk of severe COVID-19 [5]. Age categories were 12–15 years (BNT162b2 only), 16–17 (BNT162b2 only), 18–19, and then 5-year increments up to 64 years (i.e., 20–24, 25–29). The matched calendar date became the matched Time 0 for the unboosted group. Individuals matched as unboosted comparators on an eligible calendar date could subsequently receive a booster/additional dose and enter the boosted group on the date of vaccination. All eligibility criteria, covariates, and follow-up were assessed relative to Time 0 (eFig. 1).

For inclusion, individuals were required to have received a complete COVID-19 vaccine primary series (defined as receiving 2 doses of the same brand of an mRNA vaccine with the second dose received no earlier than 4 days before the recommended interval [6]—day 17 for BNT162b2, day 24 mRNA-1273—and no later than 42 days [inclusive] after the first dose; receiving 1 dose of JNJ-7836735) without receiving any other COVID-19 vaccine doses. Individuals were also required to have at least 365 days of continuous medical and pharmacy coverage in the database that began on or before the date of the brand’s initial age-specific authorization (i.e., 11 December 2020, for individuals aged ≥ 16 years, 10 May 2021, for individuals aged 12–15 years, and 29 October 2021, for individuals aged 5–11 years) to ensure capture of all primary series and booster doses. Lastly, they were also required to be aged within the brand-specific authorized age range for booster/additional doses during the study period (≥12 years for BNT162b2; ≥18 years for mRNA-1273 and JNJ-7836735) and reside within the catchment area of the combined claims and IIS data. To match unboosted individuals who were eligible for booster/additional doses on a particular date and to better align the boosted and unboosted groups, all individuals were required to be free of any of the following conditions or treatments (which may result in temporary deferment of vaccination) in the specified time periods before Time 0: monoclonal antibody or convalescent plasma treatment (90 days); COVID-19 diagnosis (30 days); fever, nausea/vomiting, rash diagnosis (3 days); hospitalization or emergency department (ED) visit (3 days). Patients who were hospitalized or long-term care residents on Time 0 were also excluded.

Individuals were followed from Time 0 until occurrence of the study outcome or the first of the following censoring criteria: end of the data source–specific study period; disenrollment from health plan; or receipt of any subsequent COVID-19 vaccine (for either exposure group).

### Exposure assessment

Vaccine doses were identified in insurance claims and IIS records using procedure billing codes for vaccine administration, National Drug Codes for vaccine products, or vaccine administered (CVX) codes [4], [7], [8]. The dose number (i.e., first or second primary series dose, booster) was inferred from the order of observed doses within an individual’s record. A booster/additional dose was considered to be any COVID-19 vaccine dose received 28 days or greater after the completion of a primary series. Brand-unspecified codes without other accompanying claims or IIS records indicating the vaccine brand were used to indicate history of vaccination, but they were not eligible to be considered as booster/additional doses.

### Outcome assessment

Recorded diagnoses of COVID-19 were identified with diagnosis codes in the claims data [9], [10], [11], [12], [13]. Two COVID-19 outcomes were identified: (1) a COVID-19 diagnosis from any medically attended setting (hospital, ED, outpatient, or physician encounters); and (2) hospital/ED-diagnosed COVID-19. For either outcome, the diagnosis code could be in any coding position, and the recorded date of the diagnosis was the outcome date.

### Covariates

Baseline characteristics of the vaccine exposure groups were measured on or before Time 0 (eFig. 1) using enrollment, diagnosis, procedure, and pharmacy data. Covariates included demographic characteristics, comorbidities, indicators of frailty, healthcare utilization, and conditions potentially putting individuals at higher risk of severe COVID-19 [5].

### Statistical analysis

The distribution of characteristics of the matched groups were described; continuous variables were described with means, standard deviations, medians, and first and third quartiles. Categorical variables were described with counts and proportions. The balance of covariates between vaccine exposure groups was evaluated with absolute standardized differences [14].

Besides matching, we used stabilized inverse probability of treatment (sIPT) weights, derived from propensity score [15], which used the matching factors and prespecified covariates (given in eTables 3–5) as independent variables to predict the probability of receiving a booster/additional dose. The matching factors were included in propensity score models to ensure continued balance of these factors after weighting. sIPT weights were truncated at the 1st and 99th percentiles to reduce the influence of extreme weights [16].

All outcome analyses were performed separately for the 2 COVID-19 outcomes. The sIPT-weighted cumulative incidence of each COVID-19 outcome was estimated as 1 minus the Kaplan-Meier estimator [17] and plotted. A hazard ratio (HR) for each outcome was estimated using an sIPT-weighted Cox proportional hazards model. The 95% confidence intervals (CIs) were estimated with robust sandwich variance estimators. Vaccine effectiveness (VE) was estimated as 1 minus the HR.

COVID-19 incidence in the first 10 days of follow-up was considered as a negative control outcome analysis, as COVID-19 vaccines—even booster doses among previously primed individuals—are not expected to have any meaningful protective effect against COVID-19 immediately after vaccination [18], [19]. The cumulative incidence curves and HRs and 95% CIs during this negative control period were evaluated.

Subgroup analyses were performed within eras of predominant circulating variants (Delta era, 1 June 2021–24 December 2021; Omicron era, 25 December 2021–end of data availability [20]): the analysis of each variant-specific era was restricted to those with Time 0 s within the era, and follow-up was censored on the last day of the era. As the timing of authorizations and recommendations for receiving an additional dose of some vaccine brands for immunocompromised individuals differed from those for booster doses in other groups [21], and because VE may differ by immunocompromised status [22], subgroup analyses were performed separately among immunocompromised and immunocompetent individuals. Immunocompromised status was defined as having at least 2 diagnostic codes for HIV, hematological malignancy, immune deficiencies, solid malignancy, or rheumatological/inflammatory condition, or at least 1 claim containing evidence an organ transplant in the 6 months before Time 0. Lastly, subgroup analyses by heterologous or homologous booster/additional dose status were performed. Homologous boosters were defined as the brand of the booster/additional dose matching that of the primary series; heterologous boosters (any other primary series) were defined as the brand of the primary series being any brand other than that of the booster/additional dose (e.g., BNT162b2 booster after a mNRA-1273 or JNJ-7836735 primary series); heterologous boosters (single-brand primary series) were defined as a specific booster brand after a specific brand of primary series (e.g., BNT162b2 booster after a mRNA-1273 primary series).

Quantitative bias analyses [23], [24] were performed to estimate the impact of individuals truly receiving a booster/additional dose being misclassified as unboosted because of missing vaccine records. Estimates of statewide receipt of a booster/additional COVID-19 vaccine dose from CDC, state departments of health, and capture-recapture methods [25], [26], [27] were obtained and compared with observed state-level estimates in the linked data sources to estimate maximum and minimum potential sensitivities of the study’s vaccine exposure measurement. HRs accounting for the minimum and maximum vaccine exposure sensitivities were estimated (Supplemental Methods); sensitivity estimates for boosted status were 69% and 84% in Optum and 69% and 89% in CVS Health.

All analyses were performed and reported separately in each database. Given that a common study protocol was applied in 2 similar, national commercial claims database that cover populations within similar demographics, database-specific results were meta-analyzed with fixed-effects meta-analysis models [28]. Statistical evidence of heterogeneity between data sources was evaluated, with *p* values less than 0.05 indicating evidence of statistical heterogeneity between study estimates.

Statistical analyses were performed with SAS Version 9.4 (SAS Institute Inc., Cary, NC). This activity was conducted as part of the FDA public health surveillance mandate, and FDA did not require Institutional Review Board review; individual-level consent was not required for this analysis of secondary healthcare data. The study protocol was publicly posted on the BEST Initiative web page [29].

## Results

We identified a total of 1,413,329 eligible individuals across both databases who received a booster/additional dose during the study period (62% BNT162b2, 37% mRNA-1273, 1% JNJ-7836735) (eFig. 2). After matching to unboosted comparators, the resulting BNT162b2 cohorts contained 118,326 matched pairs for Optum and 633,839 for CVS Health; the mRNA-1273 cohorts included 68,117 matched pairs for Optum and 342,384 for CVS Health; and the JNJ-7836735 cohorts had 1,615 matched pairs for Optum and 9,783 for CVS Health (Table 1, and eFig. 2). Selected characteristics of the matched treatment groups are shown in Table 1 (complete characteristics for all matched cohorts, by brand, are shown in eTables 3–5). For each matched cohort, the distributions of the propensity scores demonstrated a high degree of overlap, suggesting comparability between the groups (eFig. 3).

**Table 1:** Selected characteristics of individuals receiving a booster/additional dose of a COVID-19 vaccine and matched individuals with a complete primary series without a booster/additional dose.

A. BNT-162b2 booster/additional dose				
Characteristic	Optum		CVS Health	
	Individuals receiving a booster/additional dose N = 118,326	Matched unboosted individuals N = 118,326	Individuals receiving a booster/additional dose N = 633,839	Matched unboosted individuals N = 633,839
Median age, years (Q1, Q3)	43 (31, 54)	43 (31, 54)	41 (28, 53)	41 (28, 53)
Female, N (%)	61,089 (51.63%)	61,089 (51.63%)	342,679 (54.06%)	342,679 (54.06%)
Median days since primary completion (Q1, Q3)	226 (209, 246)	226 (208, 246)	231 (212, 258)	231 (211, 257)

*Primary series brand, N (%)*				
JNJ-7836735	5,026 (4.25%)	5,026 (4.25%)	20,496 (3.23%)	20,496 (3.23%)
mRNA-1273	6,336 (5.35%)	6,336 (5.35%)	29,869 (4.71%)	29,869 (4.71%)
BNT162b2	106,964 (90.40%)	106,964 (90.40%)	583,474 (92.05%)	583,474 (92.05%)

*Region, N (%)*				
Northeast	14,961 (12.64%)	14,961 (12.64%)	93,202 (14.70%)	93,202 (14.70%)
South	4,815 (4.07%)	4,815 (4.07%)	123,007 (19.41%)	123,007 (19.41%)
Midwest	68,497 (57.89%)	68,497 (57.89%)	116,633 (18.40%)	116,633 (18.40%)
West	30,053 (25.40%)	30,053 (25.40%)	300,997 (47.49%)	300,997 (47.49%)
Pregnant, N (%)	603 (0.99%)	681 (1.11%)	3,667 (0.58%)	4,009 (0.63%)
Immunocompromised, N (%)	3,725 (3.15%)	3,725 (3.15%)	18,592 (2.93%)	18,592 (2.93%)
Influenza vaccination received, N (%)	45,631 (38.56%)	45,631 (38.56%)	206,451 (32.57%)	206,451 (32.57%)

B. mRNA-1273 booster/additional dose				
Characteristic	Optum		CVS Health	
	Individuals receiving a booster/additional dose N = 68,117	Matched unboosted individuals N = 68,117	Individuals receiving a booster/additional dose N = 342,384	Matched unboosted individuals N = 342,384
Median age, years (Q1, Q3)	45 (35, 56)	45 (35, 56)	46 (34, 56)	46 (34, 56)
Female, N (%)	34,613 (50.81%)	34,613 (50.81%)	177,495 (51.84%)	177,495 (51.84%)
Median days since primary completion (Q1, Q3)	229 (211, 250)	229 (211, 250)	233 (213, 258)	232 (213, 258)

*Primary series brand, N (%)*				
JNJ-7836735	5,457 (8.01%)	5,457 (8.01%)	24,503 (7.16%)	24,503 (7.16%)
mRNA-1273	51,656 (75.83%)	51,656 (75.83%)	257,913 (75.33%)	257,913 (75.33%)
BNT162b2	11,004 (16.15%)	11,004 (16.15%)	59,968 (17.51%)	59,968 (17.51%)

*Region, N (%)*				
Northeast	8,357 (12.27%)	8,357 (12.27%)	58,817 (17.18%)	58,817 (17.18%)
South	1,959 (2.88%)	1,959 (2.88%)	43,146 (12.60%)	43,146 (12.60%)
Midwest	39,636 (58.19%)	39,636 (58.19%)	59,997 (17.52%)	59,997 (17.52%)
West	18,165 (26.67%)	18,165 (26.67%)	180,424 (52.70%)	180,424 (52.70%)
Pregnant, N (%)	393 (1.14%)	426 (1.23%)	1,749 (0.51%)	2,036 (0.59%)
Immunocompromised, N (%)	1,932 (2.84%)	1,932 (2.84%)	10,432 (3.05%)	10,432 (3.05%)
Influenza vaccination received, N (%)	26,325 (38.65%)	26,325 (38.65%)	116,297 (33.97%)	116,297 (33.97%)

C. JNJ-7836735 booster/additional dose				
Characteristic	Optum		CVS Health	
	Individuals receiving a booster/additional dose N = 1,615	Matched unboosted individuals N = 1,615	Individuals receiving a booster/additional dose N = 9,783	Matched unboosted individuals N = 9,783
Median age, years (Q1, Q3)	50 (39, 58)	50 (39, 58)	51 (40, 58)	51 (40, 58)
Female, N (%)	699 (43.28%)	699 (43.28%)	4,346 (44.42%)	4,346 (44.42%)
Median days since primary completion (Q1, Q3)	232 (214, 252)	232 (213, 252)	233 (213, 253)	233 (213, 253)
Primary series brand, N (%)				
JNJ-7836735	1,496 (92.63%)	1,496 (92.63%)	8,995 (91.95%)	8,995 (91.95%)
mRNA-1273	47 (2.91%)	47 (2.91%)	282 (2.88%)	282 (2.88%)
BNT162b2	72 (4.46%)	72 (4.46%)	506 (5.17%)	506 (5.17%)
Region, N (%)				
Northeast	191 (11.83%)	191 (11.83%)	2,173 (22.21%)	2,173 (22.21%)
South	32 (1.98%)	32 (1.98%)	1,340 (13.70%)	1,340 (13.70%)
Midwest	950 (58.82%)	950 (58.82%)	1,298 (13.27%)	1,298 (13.27%)
West	442 (27.37%)	442 (27.37%)	4,972 (50.82%)	4,972 (50.82%)
Pregnant, N (%)	0 (0.00%)	< 11	19 (0.19%)	26 (0.27%)
Immunocompromised, N (%)	37 (2.29%)	37 (2.29%)	222 (2.27%)	222 (2.27%)
Influenza vaccination received, N (%)	445 (27.55%)	445 (27.55%)	2,280 (23.31%)	2,280 (23.31%)

COVID–19 = coronavirus disease 2019; Q1, Q3 = first and third quartile.

Note: Optum privacy rules require masking cell sizes of fewer than 11 individuals.

For BNT162b2 and mRNA-1273, there was a maximum follow-up of 201 days in Optum and 232 days in CVS Health; for JNJ-7836735, the maximum follow-up was shorter (131 days, Optum; 186 days, CVS Health) given the later authorization date (eTable 6). In Optum, the median follow-up for all boosted groups was 75–85 days (depending on the vaccine and outcome); in CVS Health, median follow-up among the booster groups ranged from 107 to 119 days. Median follow-up was shorter for the unboosted comparator groups (eTable 6), as individuals in the unboosted group were frequently censored for receiving a booster/additional dose. The cumulative incidence of COVID-19 diagnoses by booster/additional dose exposure over time are shown in eFig. 4. For all vaccine comparisons and for both outcomes, the risk of COVID-19 was higher in the unboosted comparator group than in the booster/additional dose group throughout follow-up.

**Fig. 1:**
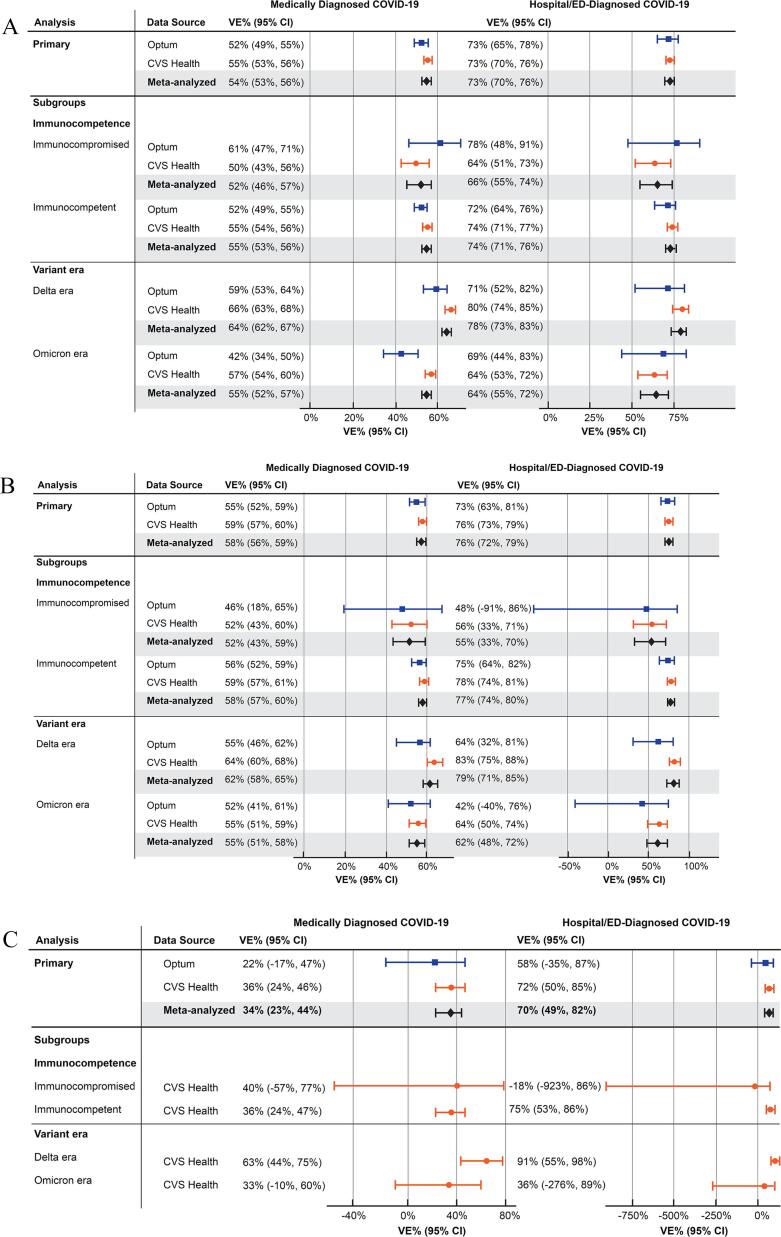
Estimated effectiveness of a booster/additional dose of COVID-19 vaccine, overall and subgroup analyses. A. BNT-162b2. B. mRNA-1273. C. JNJ-7836735. CI = confidence interval; COVID-19 = coronavirus disease 2019; ED = emergency department; VE = vaccine effectiveness.

Overall, VE estimates for all vaccines were higher against hospital/ED-diagnosed COVID-19 than against any medically diagnosed COVID-19 (Table 2; Fig. 1). For BNT162b2—the most frequently used booster and the booster with the widest age authorization during the study period—the *meta*-analyzed VE estimates were 54% (95% CI, 53%–56%) against any medically diagnosed COVID-19 and 73% (95% CI, 70%–76%) against hospital/ED-diagnosed COVID-19. Meta-analyzed VE estimates for mRNA-1273 were similarly high (medically diagnosed VE = 58% [95% CI, 56%–59%]; hospital/ED-diagnosed VE = 76% [95% CI, 72%–79%]). For JNJ–7836735, VE estimates were imprecise because of smaller sample sizes, but a higher VE against hospital/ED-diagnosed COVID-19 was still observed (medically diagnosed VE = 34% [95% CI, 23%–44%]; hospital/ED-diagnosed VE = 70% [95% CI, 49%–82%]).

**Table 2:** Estimated Effectiveness of a Booster/Additional Dose of COVID-19 Vaccine Compared With Not Receiving a Booster/Additional Dose Among Individuals Who Received a Complete Primary Series.

COVID-19 outcome	Data source	Vaccine booster/additional dose exposure group	N	Events	Person-time (days)	VE (95 % CI)^a^
*BNT162b2 booster*						
Medically diagnosed	Optum	BNT162b2	118,326	2,415	8,807,161	52% (49%, 55%)
		None	118,326	3,001	5,261,360	—
	CVS Health	BNT162b2	633,839	11,760	67,365,604	55% (53%, 56%)
		None	633,839	14,338	35,902,792	—

Hospital/ED–diagnosed	Optum	BNT162b2	118,326	113	8,916,106	73% (65%, 78%)
		None	118,326	247	5,387,213	—
	CVS Health	BNT162b2	633,839	710	68,162,932	73% (70%, 76%)
		None	633,839	1,452	36,766,210	—

*mRNA-1273 booster*						
Medically diagnosed	Optum	mRNA-1273	68,117	1,297	5,143,221	55% (52%, 59%)
		None	68,117	1,710	2,996,789	—
	CVS Health	mRNA-1273	342,384	5,722	36,334,171	59% (57%, 60%)
		None	342,384	7,763	19,323,986	—

Hospital/ED–diagnosed	Optum	mRNA-1273	68,117	59	5,201,099	73% (63%, 81%)
		None	68,117	133	3,066,068	—
	CVS Health	mRNA-1273	342,384	323	36,715,628	76% (73%, 79%)
		None	342,384	823	19,786,800	—

*JNJ-7836735 booster*						
Medically diagnosed	Optum	JNJ-7836735	1,615	58	128,604	22% (−17%, 47 %)
		None	1,615	49	81,651	—
	CVS Health	JNJ-7836735	9,783	298	1,078,067	36% (24%, 46%)
		None	9,783	285	653,948	—

Hospital/ED-diagnosed	Optum	JNJ-7836735	1,615	<11	131,182	58% (−35%, 87%)
		None	1,615	<11	83,704	—
	CVS Health	JNJ-7836735	9,783	19	1,098,347	72% (50%, 85%)
		None	9,783	41	671,553	—

CI = confidence interval; COVID–19 = coronavirus disease 2019; ED = emergency department; VE = vaccine effectiveness.

Note: indicates the reference group.

Note: Optum privacy rules require masking cell sizes of fewer than 11 individuals.

a VE estimated as 1 minus the inverse probability of treatment-weighted hazard ratio estimate.

Quantitative bias analyses correcting for 2 different potential differential misclassification scenarios suggest that the observed VE estimates may underestimate the true VE by up to 12% depending on the vaccine and outcome (eTable 7).

The differences in COVID-19 outcomes between vaccine exposure groups were evaluated during the 10-day negative control period; negative control analyses for JNJ-7836735 in Optum were underpowered due to small sample sizes and were not performed. The incidence of all COVID-19 outcomes was very low during this period (the cumulative incidence of outcomes in the unboosted comparator groups for all brands was generally around or below 0.004 for medically diagnosed COVID-19 and 0.0003 for hospital/ED-diagnosed COVID-19); the absolute differences in COVID-19 incidence between the booster/additional dose and unboosted groups were small (eFig. 5). However, the estimated VEs during the early negative control period indicated associations of vaccination with COVID-19 outcomes (eTable 8).

When stratifying the VE estimates by immunocompromised status, case counts were small for the immunocompromised group (eTable 9). The incidence rates of COVID-19 outcomes were generally higher in the immunocompromised group compared with the immunocompetent group, but for all vaccines, VE estimates for hospital/ED-diagnosed COVID-19 were generally slightly lower in the immunocompromised groups than in the immunocompetent group (e.g., for BNT162b2, *meta*-analyzed immunocompromised VE = 66% [95% CI, 55%-74%], immunocompetent VE = 74% [95% CI, 71%-76%]) (Fig. 1; eTable 9). When comparing VE within different SARS-CoV-2 variant-specific eras, VE estimates for all vaccines and outcomes were generally lower in the Omicron era than the Delta era (Fig. 1; eTable 10) (sample sizes for JNJ-7836735 were too small for meaningful subgroup analyses in Optum).

**Fig. 2:**
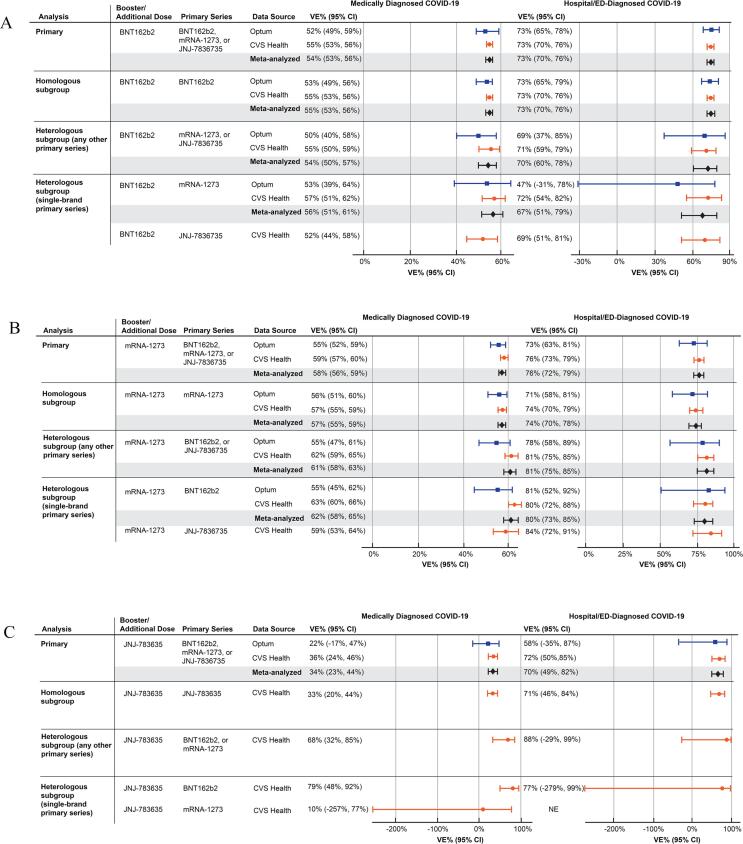
Estimated effectiveness of a booster/additional dose of COVID-19 vaccine compared with not receiving a booster/additional dose among individuals who received a complete primary series, by heterologous or homologous booster status. A. BNT162b2. B. mRNA-1273. C. JNJ-7836735. CI = confidence interval; COVID-19 = coronavirus disease 2019; ED = emergency department; VE = vaccine.

For the analyses of heterologous or homologous booster status, the majority of individuals received homologous boosters (i.e., a booster brand that matched the brand of their primary series), and sample sizes for heterologous boosters (i.e., a booster brand that did not match the brand of their primary series) were relatively small (eTable 11). For individuals receiving a BNT162b2 booster dose, there was little difference in the VE between homologous boosters and heterologous boosters; the *meta*-analyzed VE for hospital/ED-diagnosed COVID-19 was 70% (95% CI, 60%-78%) for heterologous with any non-BNT162b2 primary series and 73% (95% CI, 70%–76%) for homologous (Fig. 2). For mRNA-1273 booster recipients, VEs for heterologous boosters were similar to or slightly higher than homologous boosters (e.g., hospital/ED-diagnosed COVID-19, heterologous VE = 81%, 95% CI, 75%–85%; homologous VE = 74%, 95% CI 70%–78%). The estimates for heterologous boosters with single-brand primary series (i.e., specific combinations of booster brand and primary series brand) were similar to those for the heterologous (any other primary series) comparisons (Fig. 2).

## Discussion

This large, real-world evaluation of COVID-19 vaccine effectiveness observed lower rates of COVID-19 diagnoses in individuals who received a booster/additional dose after a complete primary series than those who received only a complete primary series but no booster/additional dose. There was also variation by vaccine brand and variant era. All booster/additional doses demonstrated stronger protective associations against hospital/ED-diagnosed COVID-19 than for any medically diagnosed COVID-19. This effectiveness appears generally sustained for at least 3–4 months after vaccination (approximately the median duration of follow-up). The boosters’ effectiveness may be sustained longer, but because of the smaller sample sizes past approximately 4 months, it is difficult to determine since estimates of the relative incidence of COVID-19 were imprecise.

The effectiveness of monovalent booster or additional doses have been evaluated in the US in a variety of settings with a variety of data sources, methodologic approaches, and study designs [22], [30], [31], [32], [33], [34], [35], [36], [37], [38], [39]. Previous studies have also demonstrated a potential waning of booster dose effectiveness over time and as viral variants have changed [30], [33], [34], [35]. The present study used national claims databases supplemented with IIS records and robust methodology to avoid time-related biases [40] while evaluating VE in different brands, subgroups, viral variant eras, and homologous and heterologous booster doses.

This study evaluated booster doses (a dose received after the completion of the primary series) and additional doses (an additional dose received as part of an extended primary series among immunocompromised individuals) [1], [2], [3] together. Operationalization of vaccine rollout, prioritization, and definitions of immunocompromised status may have differed across geography and over time, making it difficult to differentiate those receiving a booster dose and immunocompromised individuals receiving an additional dose. Subgroup analyses in immunocompromised and immunocompetent individuals demonstrated that the immunocompromised group had a higher incidence of COVID-19 outcomes, the estimated effectiveness of the vaccines against hospital/ED-diagnosed COVID-19 was slightly lower than the immunocompetent group, but the VEs for any medically diagnosed COVID-19 were generally comparable across immunocompetence groups.

The current cohort study of booster/additional doses evaluated COVID-19 outcomes in a population of individuals who had received a complete primary series. Rather than comparing boosted individuals to completely unvaccinated individuals, this comparison among individuals with complete primary series reflects the real-world decision previously vaccinated individuals face about whether to receive a booster dose. We aligned the evaluation of eligibility criteria and the start of follow-up at Time 0 in the boosted and unboosted groups; starting follow-up on Time 0 without considering future vaccination behaviors avoided immortal person-time bias [40]. Additionally, the eligibility and matching criteria were designed to only identify boosted and unboosted individuals who were eligible to receive a booster/additional dose, avoiding selection bias [40]. Vaccine booster recommendations and rollout varied over time and geography; the matching criteria were intended to identify unboosted individuals who were eligible for a booster dose, but time- or area-specific boosting eligibility may have been affected by additional factors not captured in the study data.

The current study utilized 2 data sources to include individuals from across the US combined with IIS data to be broadly representative of commercially insured individuals receiving booster/additional doses and receiving care in a variety of settings and regions during the study period. Pandemic conditions varied widely in the US over time and across geography, and thus we matched boosted and unboosted individuals on calendar day and county to account for these differences. Unboosted individuals were matched with replacement, and thus unboosted individuals could be included multiple times. However, the majority of comparator episodes were contributed by unique individuals (eFig. 2), and the dependence of multiple records from the same individuals was accounted for in the calculation of variance.

This real-world study is subject to limitations common to observational research using existing data sources. We used combined claims and IIS records to identify vaccination status, improving vaccination capture compared to either data source alone. However, some vaccines may still be missed, resulting in misclassification of vaccine exposure. This was evaluated with quantitative bias analysis, which suggested that the observed VE estimates may slightly underestimate true VE.

Studies have evaluated the effectiveness of COVID-19 vaccines against multiple outcomes, (e.g., including infection, symptomatic infection, hospitalization, death). Our study only considered COVID-19 diagnoses recorded in medical claims rather than laboratory-confirmed COVID-19 or COVID-19 infection, because neither laboratory results nor at-home test results were available in the databases. Because COVID-19 testing and diagnosis practices changed over the course of the study period with larger availability of at-home testing later in the pandemic, identifying all COVID-19 infections or symptomatic infections was not possible. Additionally, some COVID-19 diagnoses may result from mild or asymptomatic cases being identified secondary to seeking care for something else. However, ICD-10-CM diagnosis codes for COVID-19 have shown reasonable validity, particularly for hospitalized COVID-19 [9], [10], [11], [12], [13], but not all COVID-19 infections result in medical interactions and recorded diagnoses. Although asymptomatic or mild cases would not be captured by our outcome definitions, hospitalizations for COVID-19 are a meaningful metric for public health surveillance [41].

Despite matching on demographic and clinical characteristics and IPT weighting, the negative control analysis suggested a potential difference between the vaccine exposure groups during a time when vaccines are assumed to have no biologic effect (up to 10 days after vaccination while the body mounts an immune response). Although the absolute differences between the boosted and unboosted groups during this period was small, differences in COVID-19 testing, care-seeking, and diagnosis recording may have differed in boosted individuals on or immediately after vaccination compared with individuals not receiving a vaccine, as noted in other studies [42]. However, longer term differences in healthcare-seeking behavior cannot be ruled out. Despite matching and weighting methods, residual confounding by unmeasured factors may remain.

This study considered the first booster/additional dose following primary series completion, and it only considered monovalent boosters and additional doses. However, subsequent authorizations have been made for additional vaccine brands and platforms, additional rounds of boosters in select populations, expanded pediatric age groups, and updated vaccines against more recent viral variants. Thus, these results may not be applicable to future periods of the COVID-19 pandemic or updated vaccine formulations.

## Conclusions

The original, monovalent booster/additional doses were reasonably effective in real-world use in preventing medically diagnosed COVID-19 and even more effective in preventing hospital/ED-diagnosed COVID-19 among the populations for which they were indicated during the study period. Among those who had received primary series of COVID-19 vaccination, receipt of any booster/additional dose conveyed some level of protection for at least a few months. In the setting of a rapidly evolving pandemic, vaccination authorizations and recommendations have also changed rapidly; updated vaccines targeting new variants have been authorized, and vaccination schedules for updated vaccines have been modified to no longer consider a primary series and separate boosters. Additional studies and surveillance activities are needed in individuals receiving updated vaccines and in time periods with new predominant viral variants to evaluate the effectiveness of evolving vaccination strategies.

Funding sources

This work was supported by the US Food and Drug Administration.

## CRediT authorship contribution statement

**J. Bradley Layton:** Conceptualization, Investigation, Methodology, Supervision, Writing – original draft, Project administration. **Lauren Peetluk:** Data curation, Formal analysis, Methodology, Writing – review & editing. **Hui Lee Wong:** . **Yixin Jiao:** Data curation, Formal analysis, Project administration, Supervision, Writing – review & editing. **Djeneba Audrey Djibo:** Investigation, Supervision, Project administration, Writing – review & editing. **Christine Bui:** Conceptualization, Investigation, Project administration, Writing – original draft. **Patricia C. Lloyd:** Conceptualization, Investigation, Project administration, Writing – review & editing. **Joann F. Gruber:** . **Michael Miller:** Data curation, Formal analysis, Software, Writing – review & editing. **Rachel P. Ogilvie:** . **Jie Deng:** . **Ron Parambi:** Data curation, Formal analysis, Software, Writing – review & editing. **Jennifer Song:** Data curation, Formal analysis, Software, Writing – review & editing. **Lisa B. Weatherby:** Data curation, Formal analysis, Project administration, Supervision, Writing – review & editing. **An-Chi Lo:** Conceptualization, Data curation, Formal analysis, Methodology, Writing – review & editing. **Kathryn Matuska:** Conceptualization, Investigation, Project administration, Writing – review & editing. **Michael Wernecke:** . **Tainya C. Clarke:** Conceptualization, Investigation, Project administration, Writing – review & editing. **Sylvia Cho:** . **Elizabeth J. Bell:** Conceptualization, Investigation, Writing – review & editing. **John D. Seeger:** . **Grace Wenya Yang:** Conceptualization, Funding acquisition, Investigation, Supervision, Writing – review & editing. **Dóra Illei:** Data curation, Investigation, Writing – original draft. **Richard A. Forshee:** Conceptualization, Funding acquisition, Supervision, Writing – review & editing. **Steven A. Anderson:** Conceptualization, Funding acquisition, Supervision, Writing – review & editing. **Cheryl N. McMahill-Walraven:** Data curation, Formal analysis, Project administration, Supervision, Writing – review & editing. **Yoganand Chillarige:** Conceptualization, Investigation, Methodology, Project administration, Supervision, Writing – review & editing. **Kandace L. Amend:** . **Mary S. Anthony:** . **Azadeh Shoaibi:** Conceptualization, Funding acquisition, Investigation, Methodology, Project administration, Supervision, Writing – review & editing.

## Declaration of competing interest

The authors declare the following financial interests/personal relationships which may be considered as potential competing interests: JBL, WGA, ATK, CB, and MSA are employees of RTI International, an independent nonprofit institute that performs research on behalf of governmental and commercial clients, including manufacturers of COVID-19 vaccines. LP, MM, RPO, JD, RP, JS, LBW, EJB, JDS, GWY, and KLA are employees of Optum and may own stock in UnitedHealth Group. DAD and CNMW are employees of CVS Health, which performs research on behalf of governmental and commercial clients, including manufacturers of COVID-19 vaccines. All other authors have no conflicts to declare.

## Data Availability

The data that support the findings of this study are available from the respective data holders (Optum and CVS Health) but restrictions apply to the availability of these data, which were used under license for the current study, and so are not publicly available.
