# Supplementary material for: Effectiveness of monovalent COVID-19 booster/additional vaccine doses in the United States
Source: Vaccine X. 2024 Jan 20;16:100447. doi: 10.1016/j.jvacx.2024.100447 (PMC10840109; doi:10.1016/j.jvacx.2024.100447)
Supplement: Supplementary Data 1 [file mmc1.docx]

Effectiveness of Monovalent COVID-19 Booster/Additional Vaccine Doses in the United States

**ONLINE SUPPLEMENT**

J. Bradley Layton, PhD^a^

Lauren Peetluk, PhD, MPH^b^

Hui Lee Wong, PhD^c^

Yixin Jiao, MPP^d^

Djeneba Audrey Djibo, PhD^e^

Christine Bui, MPH^a^

Patricia C. Lloyd, ScM, PhD^c^

Joann F. Gruber, PhD^c^

Michael Miller, MS^b^

Rachel P. Ogilvie, PhD, MPH^b^

Jie Deng, MS^b^

Ron Parambi, MBBS, MPH^b^

Jennifer Song, MS, MURP^b^

Lisa B. Weatherby, MS^b^

An-Chi Lo, MS, MPH^d^

Kathryn Matuska, BA^d^

Michael Wernecke, BS^d^

Tainya C. Clarke, PhD^c^

Sylvia Cho, PhD^c^

Elizabeth J. Bell, PhD, MPH^b^

John D. Seeger, PharmD, DrPH^b^

Grace Wenya Yang, MPA, MA^f^

Dóra Illei, MSc^g^

Richard A. Forshee, PhD^c^

Steven A. Anderson, PhD^c^

Cheryl N. McMahill-Walraven, MSW, PhD^e^

Yoganand Chillarige, MPA^d^

Kandace L. Amend, PhD, MPH^b^

Mary S. Anthony, PhD^a^

Azadeh Shoaibi, PhD, MHS^c^

^a^ RTI Health Solutions, Research Triangle Park, NC, USA

^b^ Optum Epidemiology, Boston, MA, USA

^c^ US Food and Drug Administration, Center for Biologics Evaluation and Research, Silver Spring, MD, USA

^d^ Acumen, LLC, Burlingame, CA, USA

^e^ Safety, Surveillance & Collaboration, CVS Health, Blue Bell, PA, USA

^f^ Optum Serve, Falls Church VA, USA

^g^ RTI International, Washington, DC, USA

Source(s) of Support: US FDA

# SUPPLEMENTARY MATERIAL

## Supplemental Methods: Quantitative Bias Analysis

A simple quantitative bias analysis evaluating the impact of vaccine exposure misclassification was performed. The primary analyses estimated hazard ratios, but for the purposes of the quantitative bias analyses, risk ratios (RR) and 95% confidence intervals (CIs) were estimated in the weighted cohorts using a fixed 61-day follow-up time for both outcomes.

Specificity of the study’s vaccine assessment is assumed to be 100% (i.e., all observed claims or IIS records are assumed to be true vaccination events, and no truly unboosted individuals were misclassified as receiving a booster/additional dose). Estimates of vaccine underreporting were calculated by comparing each immunization information system (IIS) jurisdiction’s booster/additional dose receipt estimates for the population aged 65 years or younger to age-standardized booster dose receipt estimates from CDC, state health departments, and a mathematically derived booster dose coverage estimate, using capture-recapture methods [1] with IIS and claims as the 2 sources of booster dose information. Using the range of these estimates of underreporting, bounds of potential sensitivities were estimated as 69% and 84% in Optum, and 69% and 89% in CVS Health. “Corrected” RR estimates were generated for each outcome by reassigning exposure status from unboosted to boosted based on the sensitivity estimate. A correction factor was then estimated as follows:

$$bias correction factor=1-\frac{corrected RR}{uncorrected RR}$$

The bias correction factors were then applied to the observed VE estimates from the primary analyses, generating estimates of VE corrected for potential vaccine exposure misclassification.

1. IIS Jurisdictions and Study Periods Utilized

| Jurisdiction | Study period, Optum | Study Period, CVS Health |
| --- | --- | --- |
| 1 | NA | 12 August 2021 – 31 March 2022 |
| 2 | 11 December 2020 – 28 February 2022 | NA |
| 3 | NA | 12 August 2021 – 31 March 2022 |
| 4 | NA | 12 August 2021 – 31 March 2022 |
| 5 | NA | 12 August 2021 – 31 March 2022 |
| 6 | NA | 12 August 2021 – 31 March 2022 |
| 7 | NA | 12 August 2021 – 31 March 2022 |
| 8 | 11 December 2020 – 28 February 2022 | 12 August 2021 – 31 March 2022 |
| 9 | 11 December 2020 – 28 February 2022 | 12 August 2021 – 31 March 2022 |
| 10 | 11 December 2020 – 28 February 2022 | 12 August 2021 – 31 March 2022 |
| 11 | 11 December 2020 – 28 February 2022 | NA |
| 12 | 11 December 2020 – 28 February 2022 | 12 August 2021 – 31 March 2022 |
| 13 | 11 December 2020 – 28 February 2022 | NA |
| 14 | 11 December 2020 – 28 February 2022 | NA |
| 15 | 11 December 2020 – 28 February 2022 | NA |
| 16 | NA | 12 August 2021 – 31 March 2022 |

IIS = immunization information system.

1. Dates of COVID‑19 Vaccine Initial Authorization and Booster/Additional Dose Authorization by Brand in the United States During the Booster/Additional Dose Study Period, 11 December 2020 – 31 March 2022

| COVID‑19 vaccine | Number of doses in primary series | US authorization date of booster/additional doses | Authorized population |
| --- | --- | --- | --- |
| BNT162b2 | 2 | 12 August 2021 | Immunocompromised individuals |
|  |  | 22 September 2021 | Aged ≥ 65 years or at high risk of severe COVID-19 |
|  |  | 19 November 2021 | Aged ≥ 18 years |
|  |  | 3 January 2022 | Aged ≥ 12 years |
| mRNA-1273 | 2 | 12 August 2021 | Immunocompromised individuals |
|  |  | 20 October 2021 | Aged ≥ 65 years or at high risk of severe COVID-19 |
|  |  | 19 November 2021 | Aged ≥ 18 years |
| JNJ-7836735 | 1 | 20 October 2021 | Aged ≥ 18 years |

COVID‑19 = coronavirus disease 2019; FDA = Food and Drug Administration; US = United States.

Source: US FDA [2-4].

1. Characteristics of Individuals Aged 12–64 Years Who Received a BNT162b2 COVID-19 Vaccine Booster/Additional Dose and Matched Individuals Who Received a Complete Primary Series But Did Not Receive a Booster/Additional Dose

A. Optum

| Characteristic | Individuals receiving a booster/additional dose of BNT162b2  N = 118,326 | Matched, unboosted comparators  N = 118,326 | Absolute standardized difference |
| --- | --- | --- | --- |
| **Characteristics assessed at Time 0** |  |  |  |
| Age, years |  |  |  |
| Median (Q1, Q3) | 43 (31, 54) | 43 (31, 54) |  |
| Mean (SD) | 41.45 (14.75) | 41.44 (14.72) | 0.00 |
| Sex, N (%) |  |  |  |
| Male | 57,237 (48.37%) | 57,237 (48.37%) | 0.00 |
| Female | 61,089 (51.63%) | 61,089 (51.63%) | 0.00 |
| Days since primary completion |  |  |  |
| Median (Q1, Q3) | 226 (209, 246) | 226 (208, 246) |  |
| Mean (SD) | 228.10 (31.69) | 227.75 (31.88) | 0.01 |
| Primary series brand, N (%) |  |  |  |
| JNJ-7836735 | 5,026 (4.25%) | 5,026 (4.25%) | 0.00 |
| mRNA-1273 | 6,336 (5.35%) | 6,336 (5.35%) | 0.00 |
| BNT162b2 | 106,964 (90.40%) | 106,964 (90.40%) | 0.00 |
| US region, N (%) |  |  |  |
| Northeast | 14,961 (12.64%) | 14,961 (12.64%) | 0.00 |
| South | 4,815 (4.07%) | 4,815 (4.07%) | 0.00 |
| Midwest | 68,497 (57.89%) | 68,497 (57.89%) | 0.00 |
| West | 30,053 (25.40%) | 30,053 (25.40%) | 0.00 |
| Pregnant at Time 0, N (%) | 603 (0.99%) | 681 (1.11%) | 0.01 |
| **Characteristics assessed in the 365 days before Time 0** |  |  |  |
| Hospitalizations |  |  |  |
| 0 | 78,142 (66.04%) | 78,529 (66.37%) | 0.01 |
| 1 | 21,589 (18.25%) | 21,585 (18.24%) | 0.00 |
| 2+ | 18,595 (15.72%) | 18,212 (15.39%) | 0.01 |
| Emergency department visits |  |  |  |
| 0 | 107,606 (90.94%) | 106,631 (90.12%) | 0.03 |
| 1 | 9,020 (7.62%) | 9,751 (8.24%) | 0.02 |
| 2+ | 1,700 (1.44%) | 1,944 (1.64%) | 0.02 |
| Skilled nursing facility stay | 299 (0.25%) | 318 (0.27%) | 0.00 |
| Influenza vaccination | 45,631 (38.56%) | 45,631 (38.56%) | 0.00 |
| Pneumococcal vaccination | 1,474 (1.25%) | 1,308 (1.11%) | 0.01 |
| Encounter for cancer screening | 36,989 (31.26%) | 35,783 (30.24%) | 0.02 |
| Eye examination | 10,758 (9.09%) | 9,857 (8.33%) | 0.03 |
| Colonoscopy | 6,187 (5.23%) | 5,887 (4.98%) | 0.01 |
| Bone mineral density test | 1,735 (1.47%) | 1,604 (1.36%) | 0.01 |
| Well-check/well-child preventive healthcare visit | 60,497 (51.13%) | 59,055 (49.91%) | 0.02 |
| Arthritis | 17,187 (14.53%) | 17,340 (14.65%) | 0.00 |
| Lipid abnormality | 22,363 (18.90%) | 22,198 (18.76%) | 0.00 |
| Ambulance use or life support services | 1,627 (1.38%) | 1,763 (1.49%) | 0.01 |
| Weakness | 2,564 (2.17%) | 2,488 (2.10%) | 0.00 |
| Pregnancy completion before Time 0 | 1,583 (2.59%) | 1,508 (2.47%) | 0.01 |
| **Characteristics assessed using all available data, N (%)** |  |  |  |
| Autoimmune disorders | 5,637 (4.76%) | 5,338 (4.51%) | 0.01 |
| Cancer | 7,554 (6.38%) | 7,357 (6.22%) | 0.01 |
| Chronic kidney disease or renal disease | 2,269 (1.92%) | 2,255 (1.91%) | 0.00 |
| Chronic liver disease | 5,498 (4.65%) | 5,411 (4.57%) | 0.00 |
| Chronic lung diseases (e.g., asthma, COPD, cystic fibrosis, pulmonary embolism) | 14,298 (12.08%) | 13,771 (11.64%) | 0.01 |
| Dementia or other neurological conditions | 8,675 (7.33%) | 8,472 (7.16%) | 0.01 |
| Diabetes mellitus, type 1 or 2 | 9,320 (7.88%) | 9,129 (7.72%) | 0.01 |
| Down syndrome | 48 (0.04%) | 33 (0.03%) | 0.01 |
| Heart conditions (e.g., heart failure, coronary artery disease, arrhythmias) | 16,916 (14.30%) | 16,895 (14.28%) | 0.00 |
| Hypertension | 25,073 (21.19%) | 25,290 (21.37%) | 0.00 |
| Immunocompromised state | 3,725 (3.15%) | 3,725 (3.15%) | 0.00 |
| Mental health conditions | 38,183 (32.27%) | 37,749 (31.90%) | 0.01 |
| Obese or severely obese | 24,829 (20.98%) | 25,557 (21.60%) | 0.02 |
| Sickle cell disease or thalassemia | 282 (0.24%) | 285 (0.24%) | 0.00 |
| Stroke or cerebrovascular disease | 1,930 (1.63%) | 1,952 (1.65%) | 0.00 |
| Tuberculosis | 68 (0.06%) | 77 (0.07%) | 0.00 |
| At least 1 COVID-19 laboratory test performed | 56,192 (47.49%) | 56,271 (47.56%) | 0.00 |
| COVID-19 diagnoses occurring outside a hospital or emergency department setting | 6,765 (5.72%) | 7,608 (6.43%) | 0.03 |
| Hospitalization or emergency department–diagnosed COVID-19 | 899 (0.76%) | 998 (0.84%) | 0.01 |

COPD = chronic obstructive pulmonary disease; COVID‑19 = coronavirus disease 2019; Q1, Q3 = first and third quartiles; SD = standard deviation; US = United States.

B. CVS Health

| Characteristic | Individuals receiving a booster/additional dose of BNT162b2  N = 633,839 | Matched, unboosted comparators  N = 633,839 | Absolute standardized difference |
| --- | --- | --- | --- |
| **Characteristics assessed at Time 0** |  |  |  |
| Age, years |  |  |  |
| Median (Q1, Q3) | 41 (28, 53) | 41 (28, 53) | 0.00 |
| Mean (SD) | 40.22 (15.25) | 40.21 (15.21) | 0.00 |
| Sex, N (%) |  |  |  |
| Male | 291,160 (45.94%) | 291,160 (45.94%) | 0.00 |
| Female | 342,679 (54.06%) | 342,679 (54.06%) | 0.00 |
| Days since primary completion |  |  |  |
| Median (Q1, Q3) | 231 (212, 258) | 231 (211, 257) | 0.00 |
| Mean (SD) | 236.31 (37.44) | 236.09 (37.61) | 0.01 |
| Primary series brand, N (%) |  |  |  |
| JNJ-7836735 | 20,496 (3.23%) | 20,496 (3.23%) | 0.00 |
| mRNA-1273 | 29,869 (4.71%) | 29,869 (4.71%) | 0.00 |
| BNT162b2 | 583,474 (92.05%) | 583,474 (92.05%) | 0.00 |
| US region, N (%) |  |  |  |
| Northeast | 93,202 (14.70%) | 93,202 (14.70%) | 0.00 |
| South | 123,007 (19.41%) | 123,007 (19.41%) | 0.00 |
| Midwest | 116,633 (18.40%) | 116,633 (18.40%) | 0.00 |
| West | 300,997 (47.49%) | 300,997 (47.49%) | 0.00 |
| Pregnant at Time 0, N (%) | 3,667 (0.58%) | 4,009 (0.63%) | 0.01 |
| **Characteristics assessed in the 365 days before Time 0** |  |  |  |
| Hospitalizations |  |  |  |
| 0 | 443,572 (69.98%) | 491,807 (77.59%) | 0.17 |
| 1 | 100,255 (15.82%) | 85,212 (13.44%) | 0.07 |
| 2+ | 90,012 (14.20%) | 56,820 (8.96%) | 0.16 |
| Emergency department visits |  |  |  |
| 0 | 579,614 (91.44%) | 592,731 (93.51%) | 0.08 |
| 1 | 45,317 (7.15%) | 35,554 (5.61%) | 0.06 |
| 2+ | 8,908 (1.41%) | 5,554 (0.88%) | 0.05 |
| Skilled nursing facility stay | 352 (0.06%) | 300 (0.05%) | 0.00 |
| Influenza vaccination | 206,451 (32.57%) | 206,451 (32.57%) | 0.00 |
| Pneumococcal vaccination | 6,995 (1.10%) | 4,259 (0.67%) | 0.05 |
| Encounter for cancer screening | 189,088 (29.83%) | 132,812 (20.95%) | 0.21 |
| Eye examination | 72,089 (11.37%) | 46,217 (7.29%) | 0.14 |
| Colonoscopy | 31,799 (5.02%) | 19,835 (3.13%) | 0.10 |
| Bone mineral density test | 11,001 (1.74%) | 6,598 (1.04%) | 0.06 |
| Well-check/well-child preventive healthcare visit | 315,634 (49.80%) | 218,001 (34.39%) | 0.32 |
| Arthritis | 95,248 (15.03%) | 72,449 (11.43%) | 0.11 |
| Lipid abnormality | 124,567 (19.65%) | 97,636 (15.40%) | 0.11 |
| Ambulance use or life support services | 8,401 (1.33%) | 6,156 (0.97%) | 0.03 |
| Weakness | 12,544 (1.98%) | 8,646 (1.36%) | 0.05 |
| Pregnancy completion before Time 0 | 9,310 (1.47%) | 5,354 (0.84%) | 0.06 |
| **Characteristics assessed using all available data, N (%)** |  |  |  |
| Autoimmune disorders | 30,123 (4.75%) | 28,983 (4.57%) | 0.01 |
| Cancer | 39,806 (6.28%) | 39,190 (6.18%) | 0.00 |
| Chronic kidney disease or renal disease | 13,694 (2.16%) | 13,657 (2.15%) | 0.00 |
| Chronic liver disease | 32,361 (5.11%) | 33,076 (5.22%) | 0.01 |
| Chronic lung diseases (e.g., asthma, COPD, cystic fibrosis, pulmonary embolism) | 76,489 (12.07%) | 77,146 (12.17%) | 0.00 |
| Dementia or other neurological conditions | 48,030 (7.58%) | 48,879 (7.71%) | 0.01 |
| Diabetes mellitus, type 1 or 2 | 48,729 (7.69%) | 46,566 (7.35%) | 0.01 |
| Down syndrome | 304 (0.05%) | 277 (0.04%) | 0.00 |
| Heart conditions (e.g., heart failure, coronary artery disease, arrhythmias) | 92,360 (14.57%) | 94,711 (14.94%) | 0.01 |
| Hypertension | 129,629 (20.45%) | 129,594 (20.45%) | 0.00 |
| Immunocompromised state | 18,592 (2.93%) | 18,592 (2.93%) | 0.00 |
| Mental health conditions | 190,966 (30.13%) | 190,578 (30.07%) | 0.00 |
| Obese or severely obese | 128,939 (20.34%) | 132,631 (20.93%) | 0.01 |
| Sickle cell disease or thalassemia | 2,528 (0.40%) | 2,247 (0.35%) | 0.01 |
| Stroke or cerebrovascular disease | 11,343 (1.79%) | 11,775 (1.86%) | 0.01 |
| Tuberculosis | 668 (0.11%) | 707 (0.11%) | 0.00 |
| At least 1 COVID-19 laboratory test performed | 363,436 (57.34%) | 368,756 (58.18%) | 0.02 |
| COVID-19 diagnoses occurring outside a hospital or emergency department setting | 32,113 (5.07%) | 9,816 (1.55%) | 0.20 |
| Hospitalization or emergency department–diagnosed COVID-19 | 4,195 (0.66%) | 4,305 (0.68%) | 0.00 |

COPD = chronic obstructive pulmonary disease; COVID‑19 = coronavirus disease 2019; Q1, Q3 = first and third quartiles; SD = standard deviation; US = United States.

1. Characteristics of Individuals Aged 18–64 Who Received an mRNA-1273 COVID-19 Vaccine Booster/Additional Dose and Matched Individuals Who Received a Complete Primary Series But Did Not Receive a Booster/Additional Dose

A. Optum

| Characteristic | Individuals receiving a booster/additional dose of mRNA-1273  N = 68,117 | Matched, unboosted comparators  N = 68,117 | Absolute standardized difference |
| --- | --- | --- | --- |
| **Characteristics assessed at Time 0** |  |  |  |
| Age, years |  |  |  |
| Median (Q1, Q3) | 45 (35, 56) | 45 (35, 56) |  |
| Mean (SD) | 44.71 (12.46) | 44.68 (12.44) | 0.00 |
| Sex, N (%) |  |  |  |
| Male | 33,504 (49.19%) | 33,504 (49.19%) | 0.00 |
| Female | 34,613 (50.81%) | 34,613 (50.81%) | 0.00 |
| Days since primary completion |  |  |  |
| Median (Q1, Q3) | 229 (211, 250) | 229 (211, 250) |  |
| Mean (SD) | 232.31 (31.36) | 231.99 (31.60) | 0.01 |
| Primary series brand, N (%) |  |  |  |
| JNJ-7836735 | 5,457 (8.01%) | 5,457 (8.01%) | 0.00 |
| mRNA-1273 | 51,656 (75.83%) | 51,656 (75.83%) | 0.00 |
| BNT162b2 | 11,004 (16.15%) | 11,004 (16.15%) | 0.00 |
| US region, N (%) |  |  |  |
| Northeast | 8,357 (12.27%) | 8,357 (12.27%) | 0.00 |
| South | 1,959 (2.88%) | 1,959 (2.88%) | 0.00 |
| Midwest | 39,636 (58.19%) | 39,636 (58.19%) | 0.00 |
| West | 18,165 (26.67%) | 18,165 (26.67%) | 0.00 |
| Pregnant at Time 0, N (%) | 393 (1.14%) | 426 (1.23%) | 0.01 |
| **Characteristics assessed in the 365 days before Time 0** |  |  |  |
| Hospitalizations |  |  |  |
| 0 | 43,843 (64.36%) | 44,305 (65.04%) | 0.01 |
| 1 | 12,857 (18.87%) | 12,589 (18.48%) | 0.01 |
| 2+ | 11,417 (16.76%) | 11,223 (16.48%) | 0.01 |
| Emergency department visits |  |  |  |
| 0 | 62,104 (91.17%) | 61,292 (89.98%) | 0.04 |
| 1 | 5,053 (7.42%) | 5,648 (8.29%) | 0.03 |
| 2+ | 960 (1.41%) | 1,177 (1.73%) | 0.03 |
| Skilled nursing facility stay | 86 (0.13%) | 106 (0.16%) | 0.01 |
| Influenza vaccination | 26,325 (38.65%) | 26,325 (38.65%) | 0.00 |
| Pneumococcal vaccination | 945 (1.39%) | 870 (1.28%) | 0.01 |
| Encounter for cancer screening | 23,832 (34.99%) | 23,022 (33.80%) | 0.03 |
| Eye examination | 5,317 (7.81%) | 4,763 (6.99%) | 0.03 |
| Colonoscopy | 4,176 (6.13%) | 3,715 (5.45%) | 0.03 |
| Bone mineral density test | 1,183 (1.74%) | 994 (1.46%) | 0.02 |
| Well-check/well-child preventive healthcare visit | 33,498 (49.18%) | 32,554 (47.79%) | 0.03 |
| Arthritis | 10,500 (15.41%) | 10,224 (15.01%) | 0.01 |
| Lipid abnormality | 14,660 (21.52%) | 14,730 (21.62%) | 0.00 |
| Ambulance use or life support services | 900 (1.32%) | 1,031 (1.51%) | 0.02 |
| Weakness | 1,434 (2.11%) | 1,410 (2.07%) | 0.00 |
| Pregnancy completion before Time 0 | 910 (2.63%) | 879 (2.54%) | 0.01 |
| **Characteristics assessed using all available data, N (%)** |  |  |  |
| Autoimmune disorders | 3,398 (4.99%) | 3,106 (4.56%) | 0.02 |
| Cancer | 4,903 (7.20%) | 4,543 (6.67%) | 0.02 |
| Chronic kidney disease or renal disease | 1,426 (2.09%) | 1,510 (2.22%) | 0.01 |
| Chronic liver disease | 3,384 (4.97%) | 3,439 (5.05%) | 0.00 |
| Chronic lung diseases (e.g., asthma, COPD, cystic fibrosis, pulmonary embolism) | 8,190 (12.02%) | 8,073 (11.85%) | 0.01 |
| Dementia or other neurological conditions | 5,192 (7.62%) | 5,126 (7.53%) | 0.00 |
| Diabetes mellitus, type 1 or 2 | 5,714 (8.39%) | 6,273 (9.21%) | 0.03 |
| Down syndrome | 15 (0.02%) | 12 (0.02%) | 0.00 |
| Heart conditions (e.g., heart failure, coronary artery disease, arrhythmias) | 10,333 (15.17%) | 10,575 (15.52%) | 0.01 |
| Hypertension | 16,015 (23.51%) | 16,812 (24.68%) | 0.03 |
| Immunocompromised state | 1,932 (2.84%) | 1,932 (2.84%) | 0.00 |
| Mental health conditions | 22,766 (33.42%) | 21,806 (32.01%) | 0.03 |
| Obese or severely obese | 15,163 (22.26%) | 16,325 (23.97%) | 0.04 |
| Sickle cell disease or thalassemia | 144 (0.21%) | 148 (0.22%) | 0.00 |
| Stroke or cerebrovascular disease | 1,217 (1.79%) | 1,348 (1.98%) | 0.01 |
| Tuberculosis | 54 (0.08%) | 70 (0.10%) | 0.01 |
| At least 1 COVID-19 laboratory test performed | 32,244 (47.34%) | 31,739 (46.59%) | 0.01 |
| COVID-19 diagnoses occurring outside a hospital or emergency department setting | 3,845 (5.64%) | 4,464 (6.55%) | 0.04 |
| Hospitalization or emergency department–diagnosed COVID-19 | 479 (0.70%) | 619 (0.91%) | 0.02 |

COPD = chronic obstructive pulmonary disease; COVID‑19 = coronavirus disease 2019; Q1, Q3 = first and third quartiles; SD = standard deviation; US = United States.

B. CVS Health

| Characteristic | Individuals receiving a booster/additional dose of mRNA-1273  N = 342,384 | Matched, unboosted comparators N = 342,384 | Absolute standardized difference |
| --- | --- | --- | --- |
| **Characteristics assessed at Time 0** |  |  |  |
| Age, years |  |  |  |
| Median (Q1, Q3) | 46 (34, 56) | 46 (34, 56) | 0.00 |
| Mean (SD) | 44.31 (13.11) | 44.3 (13.1) | 0.00 |
| Sex, N (%) |  |  |  |
| Male | 164,889 (48.16%) | 164,889 (48.16%) | 0.00 |
| Female | 177,495 (51.84%) | 177,495 (51.84%) | 0.00 |
| Days since primary completion |  |  |  |
| Median (Q1, Q3) | 233 (213, 258) | 232 (213, 258) | 0.00 |
| Mean (SD) | 237.84 (35.54) | 237.66 (35.74) | 0.01 |
| Primary series brand, N (%) |  |  |  |
| JNJ-7836735 | 24,503 (7.16%) | 24,503 (7.16%) | 0.00 |
| mRNA-1273 | 257,913 (75.33%) | 257,913 (75.33%) | 0.00 |
| BNT162b2 | 59,968 (17.51%) | 59,968 (17.51%) | 0.00 |
| US region, N (%) |  |  |  |
| Northeast | 58,817 (17.18%) | 58,817 (17.18%) | 0.00 |
| South | 43,146 (12.60%) | 43,146 (12.60%) | 0.00 |
| Midwest | 59,997 (17.52%) | 59,997 (17.52%) | 0.00 |
| West | 180,424 (52.70%) | 180,424 (52.70%) | 0.00 |
| Pregnant at Time 0, N (%) | 1,749 (0.51%) | 2,036 (0.59%) | 0.01 |
| **Characteristics assessed in the 365 days before Time 0** |  |  |  |
| Hospitalizations |  |  |  |
| 0 | 240,125 (70.13%) | 264,602 (77.28%) | 0.16 |
| 1 | 54,795 (16.00%) | 46,803 (13.67%) | 0.07 |
| 2+ | 47,464 (13.86%) | 30,979 (9.05%) | 0.15 |
| Emergency department visits |  |  |  |
| 0 | 313,426 (91.54%) | 319,278 (93.25%) | 0.06 |
| 1 | 24,154 (7.05%) | 19,712 (5.76%) | 0.05 |
| 2+ | 4,804 (1.40%) | 3,394 (0.99%) | 0.04 |
| Skilled nursing facility stay | 223 (0.07%) | 207 (0.06%) | 0.00 |
| Influenza vaccination | 116,297 (33.97%) | 116,297 (33.97%) | 0.00 |
| Pneumococcal vaccination | 4,300 (1.26%) | 2,815 (0.82%) | 0.04 |
| Encounter for cancer screening | 114,993 (33.59%) | 81,301 (23.75%) | 0.22 |
| Eye examination | 30,968 (9.04%) | 19,988 (5.84%) | 0.12 |
| Colonoscopy | 19,955 (5.83%) | 12,508 (3.65%) | 0.10 |
| Bone mineral density test | 6,902 (2.02%) | 4,137 (1.21%) | 0.06 |
| Well-check/well-child preventive healthcare visit | 156,504 (45.71%) | 107,801 (31.49%) | 0.30 |
| Arthritis | 55,049 (16.08%) | 42,189 (12.32%) | 0.11 |
| Lipid abnormality | 81,428 (23.78%) | 65,376 (19.09%) | 0.11 |
| Ambulance use or life support services | 4,851 (1.42%) | 3,810 (1.11%) | 0.03 |
| Weakness | 6,802 (1.99%) | 4,595 (1.34%) | 0.05 |
| Pregnancy completion before Time 0 | 4,453 (1.30%) | 2,462 (0.72%) | 0.06 |
| **Characteristics assessed using all available data, N (%)** |  |  |  |
| Autoimmune disorders | 17,811 (5.20%) | 17,430 (5.09%) | 0.01 |
| Cancer | 25,132 (7.34%) | 24,341 (7.11%) | 0.01 |
| Chronic kidney disease or renal disease | 9,110 (2.66%) | 9,340 (2.73%) | 0.00 |
| Chronic liver disease | 20,365 (5.95%) | 21,399 (6.25%) | 0.01 |
| Chronic lung diseases (e.g., asthma, COPD, cystic fibrosis, pulmonary embolism) | 42,656 (12.46%) | 43,388 (12.67%) | 0.01 |
| Dementia or other neurological conditions | 27,802 (8.12%) | 28,462 (8.31%) | 0.01 |
| Diabetes mellitus, type 1 or 2 | 30,841 (9.01%) | 31,730 (9.27%) | 0.01 |
| Down syndrome | 156 (0.05%) | 130 (0.04%) | 0.00 |
| Heart conditions (e.g., heart failure, coronary artery disease, arrhythmias) | 55,913 (16.33%) | 57,605 (16.82%) | 0.01 |
| Hypertension | 83,254 (24.32%) | 85,496 (24.97%) | 0.02 |
| Immunocompromised state | 10,432 (3.05%) | 10,432 (3.05%) | 0.00 |
| Mental health conditions | 108,934 (31.82%) | 106,744 (31.18%) | 0.01 |
| Obese or severely obese | 77,750 (22.71%) | 81,662 (23.85%) | 0.03 |
| Sickle cell disease or thalassemia | 1,171 (0.34%) | 1,205 (0.35%) | 0.00 |
| Stroke or cerebrovascular disease | 7,707 (2.25%) | 8,162 (2.38%) | 0.01 |
| Tuberculosis | 374 (0.11%) | 384 (0.11%) | 0.00 |
| At least 1 COVID-19 laboratory test performed | 192,721 (56.29%) | 194,790 (56.89%) | 0.01 |
| COVID-19 diagnoses occurring outside a hospital or emergency department setting | 16,381 (4.78%) | 4,287 (1.25%) | 0.21 |
| Hospitalization or emergency department–diagnosed COVID-19 | 2,188 (0.64%) | 2,546 (0.74%) | 0.01 |

COPD = chronic obstructive pulmonary disease; COVID‑19 = coronavirus disease 2019; Q1, Q3 = first and third quartiles; SD = standard deviation; US = United States.

1. Characteristics of Individuals Aged 18–64 Years Who Received a JNJ-7836735 COVID-19 Vaccine Booster/Additional Dose and Matched Individuals Who Received a Complete Primary Series But Did Not Receive a Booster/Additional Dose

A. Optum

| Characteristic | Individuals receiving a booster/additional dose of JNJ-7836735  N = 1,615 | Matched, unboosted comparators  N = 1,615 | Absolute standardized difference |
| --- | --- | --- | --- |
| **Characteristics assessed at Time 0** |  |  |  |
| Age, years |  |  |  |
| Median (Q1, Q3) | 50 (39, 58) | 50 (39, 58) |  |
| Mean (SD) | 47.85 (11.83) | 47.71 (11.79) | 0.01 |
| Sex, N (%) |  |  |  |
| Male | 916 (56.72%) | 916 (56.72%) | 0.00 |
| Female | 699 (43.28%) | 699 (43.28%) | 0.00 |
| Days since primary completion |  |  |  |
| Median (Q1, Q3) | 232 (214, 252) | 232 (213, 252) |  |
| Mean (SD) | 232.31 (33.90) | 232.14 (33.94) | 0.00 |
| Primary series brand, N (%) |  |  |  |
| JNJ-7836735 | 1,496 (92.63%) | 1,496 (92.63%) | 0.00 |
| mRNA-1273 | 47 (2.91%) | 47 (2.91%) | 0.00 |
| BNT162b2 | 72 (4.46%) | 72 (4.46%) | 0.00 |
| US region, N (%) |  |  |  |
| Northeast | 191 (11.83%) | 191 (11.83%) | 0.00 |
| South | 32 (1.98%) | 32 (1.98%) | 0.00 |
| Midwest | 950 (58.82%) | 950 (58.82%) | 0.00 |
| West | 442 (27.37%) | 442 (27.37%) | 0.00 |
| Pregnant at Time 0, N (%) | 0 (0.00%) | < 11 | 0.11 |
| **Characteristics assessed in the 365 days before Time 0** |  |  |  |
| Hospitalizations |  |  |  |
| 0 | 1,064 (65.88%) | 1,049 (64.95%) | 0.02 |
| 1 | 303 (18.76%) | 282 (17.46%) | 0.03 |
| 2+ | 248 (15.36%) | 284 (17.59%) | 0.06 |
| Emergency department visits |  |  |  |
| 0 | 1,465 (90.71%) | 1,425 (88.24%) | 0.08 |
| 1 | 117 (7.24%) | 154 (9.54%) | 0.08 |
| 2+ | 33 (2.04%) | 36 (2.23%) | 0.01 |
| Skilled nursing facility stay | < 11 | < 11 | 0.00 |
| Influenza vaccination | 445 (27.55%) | 445 (27.55%) | 0.00 |
| Pneumococcal vaccination | 15 (0.93%) | 17 (1.05%) | 0.01 |
| Encounter for cancer screening | 576 (35.67%) | 544 (33.68%) | 0.04 |
| Eye examination | 115 (7.12%) | 128 (7.93%) | 0.03 |
| Colonoscopy | 110 (6.81%) | 98 (6.07%) | 0.03 |
| Bone mineral density test | 30 (1.86%) | 26 (1.61%) | 0.02 |
| Well-check/well-child preventive healthcare visit | 747 (46.25%) | 725 (44.89%) | 0.03 |
| Arthritis | 274 (16.97%) | 236 (14.61%) | 0.06 |
| Lipid abnormality | 406 (25.14%) | 380 (23.53%) | 0.04 |
| Ambulance use or life support services | 34 (2.11%) | 24 (1.49%) | 0.05 |
| Weakness | 32 (1.98%) | 33 (2.04%) | 0.00 |
| Pregnancy completion before Time 0 | < 11 | < 11 | 0.01 |
| **Characteristics assessed using all available data, N (%)** |  |  |  |
| Autoimmune disorders | 82 (5.08%) | 54 (3.34%) | 0.09 |
| Cancer | 126 (7.80%) | 117 (7.24%) | 0.02 |
| Chronic kidney disease or renal disease | 37 (2.29%) | 32 (1.98%) | 0.02 |
| Chronic liver disease | 94 (5.82%) | 80 (4.95%) | 0.04 |
| Chronic lung diseases (e.g., asthma, COPD, cystic fibrosis, pulmonary embolism) | 194 (12.01%) | 196 (12.14%) | 0.00 |
| Dementia or other neurological conditions | 111 (6.87%) | 107 (6.63%) | 0.01 |
| Diabetes mellitus, type 1 or 2 | 182 (11.27%) | 147 (9.10%) | 0.07 |
| Down syndrome | 0 (0.00%) | 0 (0.00%) |  |
| Heart conditions (e.g., heart failure, coronary artery disease, arrhythmias) | 259 (16.04%) | 285 (17.65%) | 0.04 |
| Hypertension | 497 (30.77%) | 480 (29.72%) | 0.02 |
| Immunocompromised state | 37 (2.29%) | 37 (2.29%) | 0.00 |
| Mental health conditions | 471 (29.16%) | 509 (31.52%) | 0.05 |
| Obese or severely obese | 445 (27.55%) | 411 (25.45%) | 0.05 |
| Sickle cell disease or thalassemia | < 11 | < 11 | 0.04 |
| Stroke or cerebrovascular disease | 34 (2.11%) | 40 (2.48%) | 0.02 |
| Tuberculosis | < 11 | < 11 | 0.00 |
| At least 1 COVID-19 laboratory test performed | 714 (44.21%) | 707 (43.78%) | 0.01 |
| COVID-19 diagnoses occurring outside a hospital or emergency department setting | 106 (6.56%) | 111 (6.87%) | 0.01 |
| Hospitalization or emergency department–diagnosed COVID-19 | 18 (1.11%) | 13 (0.80%) | 0.03 |

COPD = chronic obstructive pulmonary disease; COVID‑19 = coronavirus disease 2019; Q1, Q3 = first and third quartiles; SD = standard deviation; US = United States.

Note: privacy rules require masking cell sizes of fewer than 11 individuals.

B. CVS Health

| Characteristic | Individuals receiving a booster/additional dose of JNJ-7836735  N = 9,783 | Matched, unboosted comparators  N = 9,783 | Absolute standardized difference |
| --- | --- | --- | --- |
| **Characteristics assessed at Time 0** |  |  |  |
| Age, years |  |  |  |
| Median (Q1, Q3) | 51 (40, 58) | 51 (40, 58) | 0.00 |
| Mean (SD) | 47.65 (12.37) | 47.65 (12.39) | 0.00 |
| Sex, N (%) |  |  |  |
| Male | 5,437 (55.58%) | 5,437 (55.58%) | 0.00 |
| Female | 4,346 (44.42%) | 4,346 (44.42%) | 0.00 |
| Days since primary completion |  |  |  |
| Median (Q1, Q3) | 233 (213, 253) | 233 (213, 253) | 0.00 |
| Mean (SD) | 232.75 (36.83) | 232.67 (36.83) | 0.00 |
| Primary series brand, N (%) |  |  |  |
| JNJ-7836735 | 8,995 (91.95%) | 8,995 (91.95%) | 0.00 |
| mRNA-1273 | 282 (2.88%) | 282 (2.88%) | 0.00 |
| BNT162b2 | 506 (5.17%) | 506 (5.17%) | 0.00 |
| US region, N (%) |  |  |  |
| Northeast | 2,173 (22.21%) | 2,173 (22.21%) | 0.00 |
| South | 1,340 (13.70%) | 1,340 (13.70%) | 0.00 |
| Midwest | 1,298 (13.27%) | 1,298 (13.27%) | 0.00 |
| West | 4,972 (50.82%) | 4,972 (50.82%) | 0.00 |
| Pregnant at Time 0, N (%) | 19 (0.19%) | 26 (0.27%) | 0.01 |
| **Characteristics assessed in the 365 days before Time 0** |  |  |  |
| Hospitalizations |  |  |  |
| 0 | 7,029 (71.85%) | 7,810 (79.83%) | 0.19 |
| 1 | 1,491 (15.24%) | 1,221 (12.48%) | 0.08 |
| 2+ | 1,263 (12.91%) | 752 (7.69%) | 0.17 |
| Emergency department visits |  |  |  |
| 0 | 8,916 (91.14%) | 9,134 (93.37%) | 0.08 |
| 1 | 720 (7.36%) | 571 (5.84%) | 0.06 |
| 2+ | 147 (1.50%) | 78 (0.80%) | 0.07 |
| Skilled nursing facility stay | < 11 | < 11 | 0.01 |
| Influenza vaccination | 2,280 (23.31%) | 2,280 (23.31%) | 0.00 |
| Pneumococcal vaccination | 128 (1.31%) | 69 (0.71%) | 0.06 |
| Encounter for cancer screening | 3,305 (33.78%) | 2,293 (23.44%) | 0.23 |
| Eye examination | 851 (8.70%) | 546 (5.58%) | 0.12 |
| Colonoscopy | 618 (6.32%) | 380 (3.88%) | 0.11 |
| Bone mineral density test | 179 (1.83%) | 116 (1.19%) | 0.05 |
| Well-check/well-child preventive healthcare visit | 4,176 (42.69%) | 2,791 (28.53%) | 0.30 |
| Arthritis | 1,629 (16.65%) | 1,318 (13.47%) | 0.09 |
| Lipid abnormality | 2,626 (26.84%) | 2,099 (21.46%) | 0.13 |
| Ambulance use or life support services | 136 (1.39%) | 101 (1.03%) | 0.03 |
| Weakness | 196 (2.00%) | 137 (1.40%) | 0.05 |
| Pregnancy completion before Time 0 | 47 (0.48%) | 40 (0.41%) | 0.01 |
| **Characteristics assessed using all available data, N (%)** |  |  |  |
| Autoimmune disorders | 483 (4.94%) | 480 (4.91%) | 0.00 |
| Cancer | 764 (7.81%) | 747 (7.64%) | 0.01 |
| Chronic kidney disease or renal disease | 332 (3.39%) | 303 (3.10%) | 0.02 |
| Chronic liver disease | 610 (6.24%) | 668 (6.83%) | 0.02 |
| Chronic lung diseases (e.g., asthma, COPD, cystic fibrosis, pulmonary embolism) | 1,253 (12.81%) | 1,198 (12.25%) | 0.02 |
| Dementia or other neurological conditions | 819 (8.37%) | 829 (8.47%) | 0.00 |
| Diabetes mellitus, type 1 or 2 | 1,072 (10.96%) | 1,022 (10.45%) | 0.02 |
| Down syndrome | < 11 | 0 (0.00%) | 0.00 |
| Heart conditions (e.g., heart failure, coronary artery disease, arrhythmias) | 1,661 (16.98%) | 1,668 (17.05%) | 0.00 |
| Hypertension | 2,951 (30.16%) | 2,821 (28.84%) | 0.03 |
| Immunocompromised state | 222 (2.27%) | 222 (2.27%) | 0.00 |
| Mental health conditions | 2,821 (28.84%) | 2,883 (29.47%) | 0.01 |
| Obese or severely obese | 2,471 (25.26%) | 2,424 (24.78%) | 0.01 |
| Sickle cell disease or thalassemia | 37 (0.38%) | 20 (0.20%) | 0.03 |
| Stroke or cerebrovascular disease | 266 (2.72%) | 267 (2.73%) | 0.00 |
| Tuberculosis | < 11 | < 11 | 0.02 |
| At least 1 COVID-19 laboratory test performed | 5,134 (52.48%) | 5,401 (55.21%) | 0.05 |
| COVID-19 diagnoses occurring outside a hospital or emergency department setting | 532 (5.44%) | 205 (2.10%) | 0.18 |
| Hospitalization or emergency department–diagnosed COVID-19 | 92 (0.94%) | 85 (0.87%) | 0.01 |

COPD = chronic obstructive pulmonary disease; COVID‑19 = coronavirus disease 2019; Q1, Q3 = first and third quartiles; SD = standard deviation; US = United States.

Note: privacy rules require masking cell sizes of fewer than 11 individuals.

1. Distribution of Follow-up Time by COVID-19 Outcome, Booster/Additional Dose Status, and Data Source

| COVID-19 outcome | Booster/additional dose exposure group | N | Person-time (days) | | | |
| --- | --- | --- | --- | --- | --- | --- |
|  |  |  | Sum | Mean (SD) | Median (Q1, Q3) | Min, max |
| Optum |  |  |  |  |  |  |
| Medically diagnosed | BNT162b2 | 118,326 | 8,807,161 | 74 (37) | 75 (47, 99) | 1, 201 |
|  | None | 118,326 | 5,261,360 | 44 (33) | 38 (17, 65) | 1, 201 |
|  | mRNA-1273 | 68,117 | 5,143,221 | 76 (34) | 79 (52, 101) | 1, 201 |
|  | None | 68,117 | 2,996,789 | 44 (33) | 37 (16, 68) | 1, 201 |
|  | JNJ-7836735 | 1,615 | 128,604 | 80 (33) | 84 (57, 108) | 1, 131 |
|  | None | 1,615 | 81,651 | 51 (35) | 45 (21, 78) | 1, 131 |
| Hospital/ED-diagnosed | BNT162b2 | 118,326 | 8,916,106 | 75 (37) | 75 (48, 100) | 1, 201 |
|  | None | 118,326 | 5,387,213 | 46 (34) | 39 (18, 68) | 1, 201 |
|  | mRNA-1273 | 68,117 | 5,201,099 | 76 (34) | 80 (53, 101) | 1, 201 |
|  | None | 68,117 | 3,066,068 | 45 (33) | 38 (17, 69) | 1, 201 |
|  | JNJ-7836735 | 1,615 | 131,182 | 81 (33) | 85 (60, 108) | 1, 131 |
|  | None | 1,615 | 83,704 | 52 (35) | 47 (21, 79) | 1, 131 |
| CVS Health |  |  |  |  |  |  |
| Medically diagnosed | BNT162b2 | 633,839 | 67,365,604 | 106 (43) | 107 (79, 135) | 1, 232 |
|  | None | 633,839 | 35,902,792 | 57 (44) | 47 (18, 87) | 1, 232 |
|  | mRNA-1273 | 342,384 | 36,334,171 | 106 (36) | 112 (86, 132) | 1, 232 |
|  | None | 342,384 | 19,323,986 | 56 (44) | 45 (18, 92) | 1, 232 |
|  | JNJ-7836735 | 9,783 | 1,078,067 | 110 (36) | 118 (88, 139) | 1, 186 |
|  | None | 9,783 | 653,948 | 67 (47) | 60 (23, 109) | 1, 161 |
| Hospital/ED-diagnosed | BNT162b2 | 633,839 | 68,162,932 | 108 (42) | 108 (80, 136) | 1, 232 |
|  | None | 633,839 | 36,766,210 | 58 (44) | 49 (19, 88) | 1, 232 |
|  | mRNA-1273 | 342,384 | 36,715,628 | 107 (36) | 112 (87, 133) | 1, 232 |
|  | None | 342,384 | 19,786,800 | 58 (44) | 47 (18, 94) | 1, 232 |
|  | JNJ-7836735 | 9,783 | 1,098,347 | 112 (35) | 119 (93, 140) | 1, 186 |
|  | None | 9,783 | 671,553 | 69 (47) | 64 (25, 112) | 1, 161 |

COVID-19 = coronavirus disease 2019; ED = emergency department; min, max = minimum and maximum; Q1, Q3 = first and third quartiles.

1. Estimated Effectiveness of a Booster/Additional Dose of COVID‑19 Vaccine, Overall and Sensitivity Analyses Accounting for Potentially Missing Vaccine Records Resulting in Exposure Misclassification

| Data Source | Hypothesized sensitivity of vaccination exposure measurement | VE (95% CI) | |
| --- | --- | --- | --- |
|  |  | Medically diagnosed COVID-19 | Hospital/ED-diagnosed COVID-19 |
| **BNT162b2** |  |  |  |
| Optum | 100% (original, uncorrected) | 52% (49%, 55%) | 73% (65%, 78%) |
|  | 84% | 56% (53%, 59%) | 77% (70%, 81%) |
|  | 69% | 63% (61%, 66%) | 82% (77%, 86%) |
| CVS Health | 100% (original, uncorrected) | 55% (53%, 56%) | 73% (70%, 76%) |
|  | 89% | 57% (56%, 59%) | 75% (73%, 78%) |
|  | 69% | 67% (66%, 68%) | 82% (81%, 84%) |
| **mRNA-1273** |  |  |  |
| Optum | 100% (original, uncorrected) | 55% (52%, 59%) | 73% (63%, 81%) |
|  | 84% | 59% (56%, 63%) | 77% (68%, 84%) |
|  | 69% | 66% (64%, 69%) | 82% (76%, 88%) |
| CVS Health | 100% (original, uncorrected) | 59% (57%, 60%) | 76% (73%, 79%) |
|  | 89% | 61% (59%, 62%) | 78% (75%, 81%) |
|  | 69% | 70% (68%, 71%) | 85% (82%, 87%) |
| **JNJ-7836735** |  |  |  |
| Optum | 100% (original, uncorrected) | 22% (-17%, 47%) | 58% (-35%, 87%) |
|  | 84% | 23% (-16%, 48%) | 58% (-34%, 87%) |
|  | 69% | 25% (-12%, 49%) | 59% (-30%, 87%) |
| CVS Health | 100% (original, uncorrected) | 36% (24%, 46%) | 72% (50%, 85%) |
|  | 89% | 38% (27%, 48%) | 75% (54%, 86%) |
|  | 69% | 47% (37%, 56%) | 82% (68%, 90%) |

CI = confidence interval; COVID‑19 = coronavirus disease 2019; ED = emergency department; VE = vaccine effectiveness.

1. Estimated Effectiveness of Receiving a Booster/Additional Dose of COVID‑19 Vaccine, Time 0 Through Day 9, Negative Control Outcome Analysis

| COVID‑19 outcome | Booster/additional dose exposure group | N | Events | Person-time (days) | HR (95% CI) | VE (95% CI) |
| --- | --- | --- | --- | --- | --- | --- |
| **Optum** |  |  |  |  |  |  |
| Medically diagnosed | BNT162b2 | 118,326 | 400 | 1,165,612 | 0.65 (0.57, 0.74) | 35% (26%, 43%) |
|  | None | 118,326 | 584 | 1,107,741 | — | — |
|  | mRNA-1273 | 68,117 | 195 | 671,236 | 0.58 (0.49, 0.70) | 42% (30%, 51%) |
|  | None | 68,117 | 312 | 635,304 | — | — |
| Hospital/ED–diagnosed | BNT162b2 | 118,326 | 15 | 1,167,241 | 0.35 (0.19, 0.63) | 65% (37%, 81%) |
|  | None | 118,326 | 42 | 1,110,208 | — | — |
|  | mRNA-1273 | 68,117 | < 11 | 672,041 | 0.52 (0.24, 1.15) | 48% (-15%, 76%) |
|  | None | 68,117 | 18 | 636,622 | — | — |
| **CVS Health** |  |  |  |  |  |  |
| Medically diagnosed | BNT162b2 | 633,839 | 1,332 | 6,295,203 | 0.46 (0.43, 0.49) | 54% (51%, 57%) |
|  | None | 633,839 | 2,656 | 5,948,978 | — | — |
|  | mRNA-1273 | 342,384 | 721 | 3,403,134 | 0.49 (0.44, 0.54) | 51% (46%, 56%) |
|  | None | 342,384 | 1,335 | 3,212,054 | — | — |
|  | JNJ-7836735 | 9,783 | 30 | 97,295 | 0.73 (0.45, 1.19) | 27% (-19%, 55%) |
|  | None | 9,783 | 38 | 93,085 | — | — |
| Hospital/ED–diagnosed | BNT162b2 | 633,839 | 88 | 6,300,227 | 0.36 (0.28, 0.47) | 64% (53%, 72%) |
|  | None | 633,839 | 220 | 5,960,018 | — | — |
|  | mRNA-1273 | 342,384 | 42 | 3,405,907 | 0.28 (0.19, 0.40) | 72% (60%, 81%) |
|  | None | 342,384 | 133 | 3,217,373 | — | — |
|  | JNJ-7836735 | 9,783 | < 11 | 97,414 | 0.35 (0.07, 1.82) | 65% (-82%, 93%) |
|  | None | 9,783 | < 11 | 93,247 | — | — |

CI = confidence interval; COVID‑19 = coronavirus disease 2019; ED = emergency department; HR = hazard ratio; VE = vaccine effectiveness.

Note: — indicates the reference group.

Note: privacy rules require masking cell sizes of fewer than 11 individuals.

1. Estimated Effectiveness of Receiving a Booster/Additional Dose of COVID‑19 Vaccine, by Immunocompromised Status

| Immunocompetence status | COVID‑19 outcome | Booster/ additional dose exposure group | N | Events | Person-time (days) | HR (95% CI) | VE (95% CI) |
| --- | --- | --- | --- | --- | --- | --- | --- |
| Optum |  |  |  |  |  |  |  |
| Immunocompromised | Medically diagnosed | BNT162b2 | 3,725 | 101 | 363,450 | 0.39 (0.29, 0.53) | 61% (47%, 71%) |
|  |  | None | 3,725 | 131 | 196,333 | — | — |
|  |  | mRNA-1273 | 1,932 | 61 | 182,469 | 0.54 (0.35, 0.82) | 46% (18%, 65%) |
|  |  | None | 1,932 | 54 | 97,367 | — | — |
|  | Hospital/ED–diagnosed | BNT162b2 | 3,725 | < 11 | 368,189 | 0.22 (0.09, 0.52) | 78% (48%, 91%) |
|  |  | None | 3,725 | 18 | 202,106 | — | — |
|  |  | mRNA-1273 | 1,932 | < 11 | 185,269 | 0.52 (0.14, 1.91) | 48% (-91%, 86%) |
|  |  | None | 1,932 | < 11 | 99,630 | — | — |
| Immunocompetent | Medically diagnosed | BNT162b2 | 114,601 | 2,314 | 8,443,711 | 0.48 (0.45, 0.51) | 52% (49%, 55%) |
|  |  | None | 114,601 | 2,870 | 5,065,027 | — | — |
|  |  | mRNA-1273 | 66,185 | 1,236 | 4,960,752 | 0.44 (0.41, 0.48) | 56% (52%, 59%) |
|  |  | None | 66,185 | 1,656 | 2,899,422 | — | — |
|  | Hospital/ED–diagnosed | BNT162b2 | 114,601 | 105 | 8,547,917 | 0.28 (0.22, 0.36) | 72% (64%, 78%) |
|  |  | None | 114,601 | 229 | 5,185,107 | — | — |
|  |  | mRNA-1273 | 66,185 | 52 | 5,015,830 | 0.25 (0.18, 0.36) | 75% (64%, 82%) |
|  |  | None | 66,185 | 127 | 2,966,438 | — | — |
| CVS Health |  |  |  |  |  |  |  |
| Immunocompromised | Medically diagnosed | BNT162b2 | 18,592 | 688 | 2,353,434 | 0.50 (0.44, 0.57) | 50% (43%, 56%) |
|  |  | None | 18,592 | 643 | 1,133,288 | — | — |
|  |  | mRNA-1273 | 10,432 | 334 | 1,245,563 | 0.48 (0.40, 0.57) | 52% (43%, 60%) |
|  |  | None | 10,432 | 343 | 606,156 | — | — |
|  |  | JNJ-7836735 | 222 | < 11 | 25,470 | 0.60 (0.23, 1.57) | 40% (-57%, 77%) |
|  |  | None | 222 | < 11 | 14,328 | — | — |
|  | Hospital/ED–diagnosed | BNT162b2 | 18,592 | 99 | 2,397,791 | 0.36 (0.27, 0.49) | 64% (51%, 73%) |
|  |  | None | 18,592 | 129 | 1,170,346 | — | — |
|  |  | mRNA-1273 | 10,432 | 46 | 1,267,064 | 0.44 (0.29, 0.67) | 56% (33%, 71%) |
|  |  | None | 10,432 | 54 | 626,175 | — | — |
|  |  | JNJ-7836735 | 222 | < 11 | 25,976 | 1.18 (0.14, 10.23) | -18% (-923%, 86%) |
|  |  | None | 222 | < 11 | 14,795 | — | — |
| Immunocompetent | Medically diagnosed | BNT162b2 | 615,247 | 11,072 | 65,012,170 | 0.45 (0.44, 0.46) | 55% (54%, 56%) |
|  |  | None | 615,247 | 13,695 | 34,769,504 | — | — |
|  |  | mRNA-1273 | 331,952 | 5,388 | 35,088,608 | 0.41 (0.39, 0.43) | 59% (57%, 61%) |
|  |  | None | 331,952 | 7,420 | 18,717,830 | — | — |
|  |  | JNJ-7836735 | 9,561 | 289 | 1,052,597 | 0.64 (0.53, 0.76) | 36% (24%, 47%) |
|  |  | None | 9,561 | 277 | 639,620 | — | — |
|  | Hospital/ED–diagnosed | BNT162b2 | 615,247 | 611 | 65,765,141 | 0.26 (0.23, 0.29) | 74% (71%, 77%) |
|  |  | None | 615,247 | 1,323 | 35,595,864 | — | — |
|  |  | mRNA-1273 | 331,952 | 277 | 35,448,564 | 0.22 (0.19, 0.26) | 78% (74%, 81%) |
|  |  | None | 331,952 | 769 | 19,160,625 | — | — |
|  |  | JNJ-7836735 | 9,561 | 17 | 1,072,371 | 0.25 (0.14, 0.47) | 75% (53%, 86%) |
|  |  | None | 9,561 | 40 | 656,758 | — | — |

CI = confidence interval; COVID‑19 = coronavirus disease 2019; ED = emergency department; HR = hazard ratio; VE = vaccine effectiveness.

Note: — indicates the reference group.

Note: privacy rules require masking cell sizes of fewer than 11 individuals.

Note: JNJ-7836735 subgroup analyses not performed in Optum due to low sample sizes.

1. Estimated Effectiveness of Receiving a Booster/Additional Dose of COVID‑19 Vaccine, by Viral Variant Era

| Variant era | COVID‑19 outcome | Booster/additional dose exposure group | N | Events | Person-time (days) | HR (95% CI) | VE (95% CI) |
| --- | --- | --- | --- | --- | --- | --- | --- |
| Optum |  |  |  |  |  |  |  |
| Delta | Medically diagnosed | BNT162b2 | 84,690 | 452 | 2,820,646 | 0.41 (0.36, 0.47) | 59% (53%, 64%) |
|  |  | None | 84,690 | 805 | 2,133,612 | — | — |
|  |  | mRNA-1273 | 53,575 | 260 | 1,602,038 | 0.45 (0.38, 0.54) | 55% (46%, 62%) |
|  |  | None | 53,575 | 422 | 1,206,185 | — | — |
|  | Hospital/ED–diagnosed | BNT162b2 | 84,690 | 24 | 2,826,838 | 0.29 (0.18, 0.48) | 71% (52%, 82%) |
|  |  | None | 84,690 | 57 | 2,144,976 | — | — |
|  |  | mRNA-1273 | 53,575 | 17 | 1,605,500 | 0.36 (0.19, 0.68) | 64% (32%, 81%) |
|  |  | None | 53,575 | 35 | 1,211,270 | — | — |
| Omicron | Medically diagnosed | BNT162b2 | 33,636 | 379 | 1,414,884 | 0.58 (0.50, 0.66) | 42% (34%, 50%) |
|  |  | None | 33,636 | 584 | 1,151,232 | — | — |
|  |  | mRNA-1273 | 14,542 | 147 | 642,360 | 0.48 (0.39, 0.59) | 52% (41%, 61%) |
|  |  | None | 14,542 | 267 | 531,647 | — | — |
|  | Hospital/ED–diagnosed | BNT162b2 | 33,636 | 15 | 1,429,104 | 0.31 (0.17, 0.56) | 69% (44%, 83%) |
|  |  | None | 33,636 | 45 | 1,171,042 | — | — |
|  |  | mRNA-1273 | 14,542 | < 11 | 647,670 | 0.58 (0.24, 1.40) | 42% (-40%, 76%) |
|  |  | None | 14,542 | 12 | 541,166 | — | — |
| CVS Health |  |  |  |  |  |  |  |
| Delta | Medically diagnosed | BNT162b2 | 419,532 | 1,563 | 15,499,273 | 0.34 (0.32, 0.37) | 66% (63%, 68%) |
|  |  | None | 419,532 | 2,902 | 10,945,112 | — | — |
|  |  | mRNA-1273 | 250,665 | 727 | 7,502,444 | 0.36 (0.32, 0.40) | 64% (60%, 68%) |
|  |  | None | 250,665 | 1,351 | 5,588,962 | — | — |
|  |  | JNJ-7836735 | 7,674 | 42 | 251,764 | 0.37 (0.25, 0.56) | 63% (44%, 75%) |
|  |  | None | 7,674 | 77 | 198,450 | — | — |
|  | Hospital/ED–diagnosed | BNT162b2 | 419,532 | 98 | 15,520,422 | 0.20 (0.15, 0.26) | 80% (74%, 85%) |
|  |  | None | 419,532 | 310 | 10,987,676 | — | — |
|  |  | mRNA-1273 | 250,665 | 36 | 7,512,304 | 0.17 (0.12, 0.25) | 83% (75%, 88%) |
|  |  | None | 250,665 | 157 | 5,605,491 | — | — |
|  |  | JNJ-7836735 | 7,674 | < 11 | 252,343 | 0.09 (0.02, 0.45) | 91% (55%, 98%) |
|  |  | None | 7,674 | 14 | 199,204 | — | — |
| Omicron | Medically diagnosed | BNT162b2 | 214,307 | 1,736 | 14,698,293 | 0.43 (0.40, 0.46) | 57% (54%, 60%) |
|  |  | None | 214,307 | 3,339 | 11,089,811 | — | — |
|  |  | mRNA-1273 | 91,719 | 903 | 6,597,858 | 0.45 (0.41, 0.49) | 55% (51%, 59%) |
|  |  | None | 91,719 | 1,668 | 5,105,313 | — | — |
|  |  | JNJ-7836735 | 2,109 | 29 | 147,763 | 0.67 (0.40, 1.10) | 33% (-10%, 60%) |
|  |  | None | 2,109 | 38 | 125,888 | — | — |
|  | Hospital/ED–diagnosed | BNT162b2 | 214,307 | 110 | 14,798,731 | 0.36 (0.28, 0.47) | 64% (53%, 72%) |
|  |  | None | 214,307 | 255 | 11,266,005 | — | — |
|  |  | mRNA-1273 | 91,719 | 60 | 6,651,335 | 0.36 (0.26, 0.50) | 64% (50%, 74%) |
|  |  | None | 91,719 | 145 | 5,195,674 | — | — |
|  |  | JNJ-7836735 | 2,109 | < 11 | 149,583 | 0.64 (0.11, 3.76) | 36% (-276%, 89%) |
|  |  | None | 2,109 | < 11 | 127,897 | — | — |

CI = confidence interval; COVID‑19 = coronavirus disease 2019; ED = emergency department; HR = hazard ratio; VE = vaccine effectiveness.

Note: — indicates the reference group.

Note: privacy rules require masking cell sizes of fewer than 11 individuals.

1. Estimated Effectiveness of Receiving a Booster/Additional Dose of COVID‑19 Vaccine, by Homologous or Heterologous Primary Series Status

A. Optum

| COVID‑19 outcome | Booster/ additional dose exposure group | Primary series | N | Events | Person-time (days) | HR (95% CI) | VE (95% CI) |
| --- | --- | --- | --- | --- | --- | --- | --- |
| Homologous booster^a^ |  |  |  |  |  |  |  |
| Medically diagnosed | BNT162b2 | BNT162b2 | 106,964 | 2,182 | 7,963,968 | 0.47 (0.44, 0.51) | 53% (49%, 56%) |
|  | None | BNT162b2 | 106,964 | 2,704 | 4,731,389 | — | — |
|  | mRNA-1273 | mRNA-1273 | 51,656 | 984 | 3,934,742 | 0.44 (0.40, 0.49) | 56% (51%, 60%) |
|  | None | mRNA-1273 | 51,656 | 1,280 | 2,266,346 | — | — |
| Hospital/ED–diagnosed | BNT162b2 | BNT162b2 | 106,964 | 100 | 8,062,247 | 0.27 (0.21, 0.35) | 73% (65%, 79%) |
|  | None | BNT162b2 | 106,964 | 220 | 4,844,752 | — | — |
|  | mRNA-1273 | mRNA-1273 | 51,656 | 45 | 3,978,840 | 0.29 (0.19, 0.42) | 71% (58%, 81%) |
|  | None | mRNA-1273 | 51,656 | 93 | 2,318,115 | — | — |
| Heterologous booster (any other primary series)^b^ |  |  |  |  |  |  |  |
| Medically diagnosed | BNT162b2 | mRNA-1273 or JNJ-7836735 | 11,362 | 233 | 843,193 | 0.50 (0.42, 0.60) | 50% (40%, 58%) |
|  | None | mRNA-1273 or JNJ-7836735 | 11,362 | 297 | 529,971 | — | — |
|  | mRNA-1273 | BNT162b2 or JNJ-7836735 | 16,461 | 313 | 1,208,479 | 0.45 (0.39, 0.53) | 55% (47%, 61%) |
|  | None | BNT162b2 or JNJ-7836735 | 16,461 | 430 | 730,443 | — | — |
| Hospital/ED–diagnosed | BNT162b2 | mRNA-1273 or JNJ-7836735 | 11,362 | 13 | 853,859 | 0.31 (0.15, 0.63) | 69% (37%, 85%) |
|  | None | mRNA-1273 or JNJ-7836735 | 11,362 | 27 | 542,461 | — | — |
|  | mRNA-1273 | BNT162b2 or JNJ-7836735 | 16,461 | 14 | 1,222,259 | 0.22 (0.11, 0.42) | 78% (58%, 89%) |
|  | None | BNT162b2 or JNJ-7836735 | 16,461 | 40 | 747,953 | — | — |
| Heterologous Booster (single-brand primary series)^c^ |  |  |  |  |  |  |  |
| Medically diagnosed | BNT162b2 | mRNA-1273 | 6,336 | 107 | 444,654 | 0.47 (0.36, 0.61) | 53% (39%, 64%) |
|  | None | mRNA-1273 | 6,336 | 140 | 269,940 | — | — |
|  | mRNA-1273 | BNT162b2 | 11,004 | 199 | 766,967 | 0.45 (0.38, 0.55) | 55% (45%, 62%) |
|  | None | BNT162b2 | 11,004 | 274 | 457,615 | — | — |
| Hospital/ED–diagnosed | BNT162b2 | mRNA-1273 | 6,336 | < 11 | 449,278 | 0.53 (0.22, 1.31) | 47% (-31%, 78%) |
|  | None | mRNA-1273 | 6,336 | < 11 | 275,563 | — | — |
|  | mRNA-1273 | BNT162b2 | 11,004 | < 11 | 775,891 | 0.19 (0.08, 0.48) | 81% (52%, 92%) |
|  | None | BNT162b2 | 11,004 | 20 | 468,519 | — | — |

CI = confidence interval; COVID‑19 = coronavirus disease 2019; ED = emergency department; HR = hazard ratio; VE = vaccine effectiveness.

Note: — indicates the reference group.

^a^ Homologous booster/additional doses are of the same brand as the primary series received by the individual.

^b^ Exposure groups defined by the brand of the primary series being any other available vaccine brand other than that of the primary series.

^c^ Exposure groups defined by having received a specific brand of primary series.

Note: privacy rules require masking cell sizes of fewer than 11 individuals.

Note: JNJ-7836735 analyses not performed due to small sample sizes.

B. CVS Health

| COVID‑19 outcome | Booster/ additional dose exposure group | Primary series | N | Events | Person-time (days) | HR (95% CI) | VE (95% CI) |
| --- | --- | --- | --- | --- | --- | --- | --- |
| Homologous booster^a^ |  |  |  |  |  |  |  |
| Medically diagnosed | BNT162b2 | BNT162b2 | 583,474 | 10,835 | 62,132,093 | 0.45 (0.44, 0.47) | 55% (53%, 56%) |
|  | None | BNT162b2 | 583,474 | 13,117 | 32,932,878 | — | — |
|  | mRNA-1273 | mRNA-1273 | 257,913 | 4,393 | 27,416,278 | 0.43 (0.41, 0.45) | 57% (55%, 59%) |
|  | None | mRNA-1273 | 257,913 | 5,740 | 14,468,540 | — | — |
|  | JNJ-7836735 | JNJ-7836735 | 8,995 | 286 | 993,948 | 0.67 (0.56, 0.80) | 33% (20%, 44%) |
|  | None | JNJ-7836735 | 8,995 | 265 | 609,433 | — | — |
| Hospital/ED–diagnosed | BNT162b2 | BNT162b2 | 583,474 | 652 | 62,867,496 | 0.27 (0.24, 0.30) | 73% (70%, 76%) |
|  | None | BNT162b2 | 583,474 | 1,329 | 33,723,241 | — | — |
|  | mRNA-1273 | mRNA-1273 | 257,913 | 249 | 27,710,788 | 0.26 (0.21, 0.30) | 74% (70%, 79%) |
|  | None | mRNA-1273 | 257,913 | 590 | 14,810,757 | — | — |
|  | JNJ-7836735 | JNJ-7836735 | 8,995 | 18 | 1,013,407 | 0.29 (0.16, 0.54) | 71% (46%, 84%) |
|  | None | JNJ-7836735 | 8,995 | 37 | 625,846 | — | — |
| Heterologous booster (any other primary series)^b^ |  |  |  |  |  |  |  |
| Medically diagnosed | BNT162b2 | mRNA-1273 or JNJ-7836735 | 50,365 | 925 | 5,233,511 | 0.45 (0.41, 0.50) | 55% (50%, 59%) |
|  | None | mRNA-1273 or JNJ-7836735 | 50,365 | 1,221 | 2,969,914 | — | — |
|  | mRNA-1273 | BNT162b2 or JNJ-7836735 | 84,471 | 1,329 | 8,917,893 | 0.38 (0.35, 0.41) | 62% (59%, 65%) |
|  | None | BNT162b2 or JNJ-7836735 | 84,471 | 2,023 | 4,855,446 | — | — |
|  | JNJ-7836735 | BNT162b2 or mRNA-1273 | 788 | 12 | 84,119 | 0.32 (0.15, 0.68) | 68% (32%, 85%) |
|  | None | BNT162b2 or mRNA-1273 | 788 | 20 | 44,515 | — | — |
| Hospital/ED–diagnosed | BNT162b2 | mRNA 1273 or JNJ-7836735 | 50,365 | 58 | 5,295,436 | 0.29 (0.21, 0.41) | 71% (59%, 79%) |
|  | None | mRNA 1273 or JNJ-7836735 | 50,365 | 123 | 3,042,969 | — | — |
|  | mRNA-1273 | BNT162b2 or JNJ-7836735 | 84,471 | 74 | 9,004,840 | 0.19 (0.15, 0.25) | 81% (75%, 85%) |
|  | None | BNT162b2 or JNJ-7836735 | 84,471 | 233 | 4,976,043 | — | — |
|  | JNJ-7836735 | BNT162b2 or mRNA-1273 | 788 | < 11 | 84,940 | 0.12 (0.01, 1.29) | 88% (-29%, 99%) |
|  | None | BNT162b2 or mRNA-1273 | 788 | < 11 | 45,707 | — | — |
| Heterologous Booster (single-brand primary series)^c^ |  |  |  |  |  |  |  |
| Medically diagnosed | BNT162b2 | mRNA-1273 | 29,869 | 514 | 3,045,525 | 0.43 (0.38, 0.49) | 57% (51%, 62%) |
|  | None | mRNA-1273 | 29,869 | 694 | 1,670,124 | — | — |
|  | BNT162b2 | JNJ-7836735 | 20,496 | 411 | 2,187,986 | 0.48 (0.42, 0.56) | 52% (44%, 58%) |
|  | None | JNJ-7836735 | 20,496 | 527 | 1,299,790 | — | — |
|  | mRNA-1273 | BNT162b2 | 59,968 | 901 | 6,189,658 | 0.37 (0.34, 0.40) | 63% (60%, 66%) |
|  | None | BNT162b2 | 59,968 | 1,396 | 3,286,651 | — | — |
|  | mRNA-1273 | JNJ-7836735 | 24,503 | 428 | 2,728,235 | 0.41 (0.36, 0.47) | 59% (53%, 64%) |
|  | None | JNJ-7836735 | 24,503 | 627 | 1,568,795 | — | — |
|  | JNJ-7836735 | BNT162b2 | 506 | < 11 | 53,993 | 0.21 (0.08, 0.52) | 79% (48%, 92%) |
|  | None | BNT162b2 | 506 | 16 | 29,132 | — | — |
|  | JNJ-7836735 | mRNA-1273 | 282 | < 11 | 30,126 | 0.90 (0.23, 3.57) | 10% (-257%, 77%) |
|  | None | mRNA-1273 | 282 | < 11 | 15,383 | — | — |
| Hospital/ED–diagnosed | BNT162b2 | mRNA-1273 | 29,869 | 28 | 3,079,930 | 0.28 (0.18, 0.46) | 72% (54%, 82%) |
|  | None | mRNA-1273 | 29,869 | 62 | 1,711,440 | — | — |
|  | BNT162b2 | JNJ-7836735 | 20,496 | 30 | 2,215,506 | 0.31 (0.19, 0.49) | 69% (51%, 81%) |
|  | None | JNJ-7836735 | 20,496 | 61 | 1,331,529 | — | — |
|  | mRNA-1273 | BNT162b2 | 59,968 | 57 | 6,247,428 | 0.20 (0.15, 0.28) | 80% (72%, 85%) |
|  | None | BNT162b2 | 59,968 | 167 | 3,367,860 | — | — |
|  | mRNA-1273 | JNJ-7836735 | 24,503 | 17 | 2,757,412 | 0.16 (0.09, 0.28) | 84% (72%, 91%) |
|  | None | JNJ-7836735 | 24,503 | 66 | 1,608,183 | — | — |
|  | JNJ-7836735 | BNT162b2 | 506 | < 11 | 54,428 | 0.23 (0.01, 3.79) | 77% (-279%, 99%) |
|  | None | BNT162b2 | 506 | < 11 | 30,239 | — | — |
|  | JNJ-7836735 | mRNA-1273 | 282 | 0 | 30,512 | 0.00 (0.00, 0.00) | 100% (100%, 100%) |
|  | None | mRNA-1273 | 282 | < 11 | 15,468 | — | — |

CI = confidence interval; COVID‑19 = coronavirus disease 2019; ED = emergency department; HR = hazard ratio; VE = vaccine effectiveness.

Note: — indicates the reference group.

^a^ Homologous booster/additional doses are of the same brand as the primary series received by the individual.

^b^ Exposure groups defined by the brand of the primary series being any other available vaccine brand other than that of the primary series.

^c^ Exposure groups defined by having received a specific brand of primary series.

Note: privacy rules require masking cell sizes of fewer than 11 individuals.

1. Study Design and Variable Assessment Periods


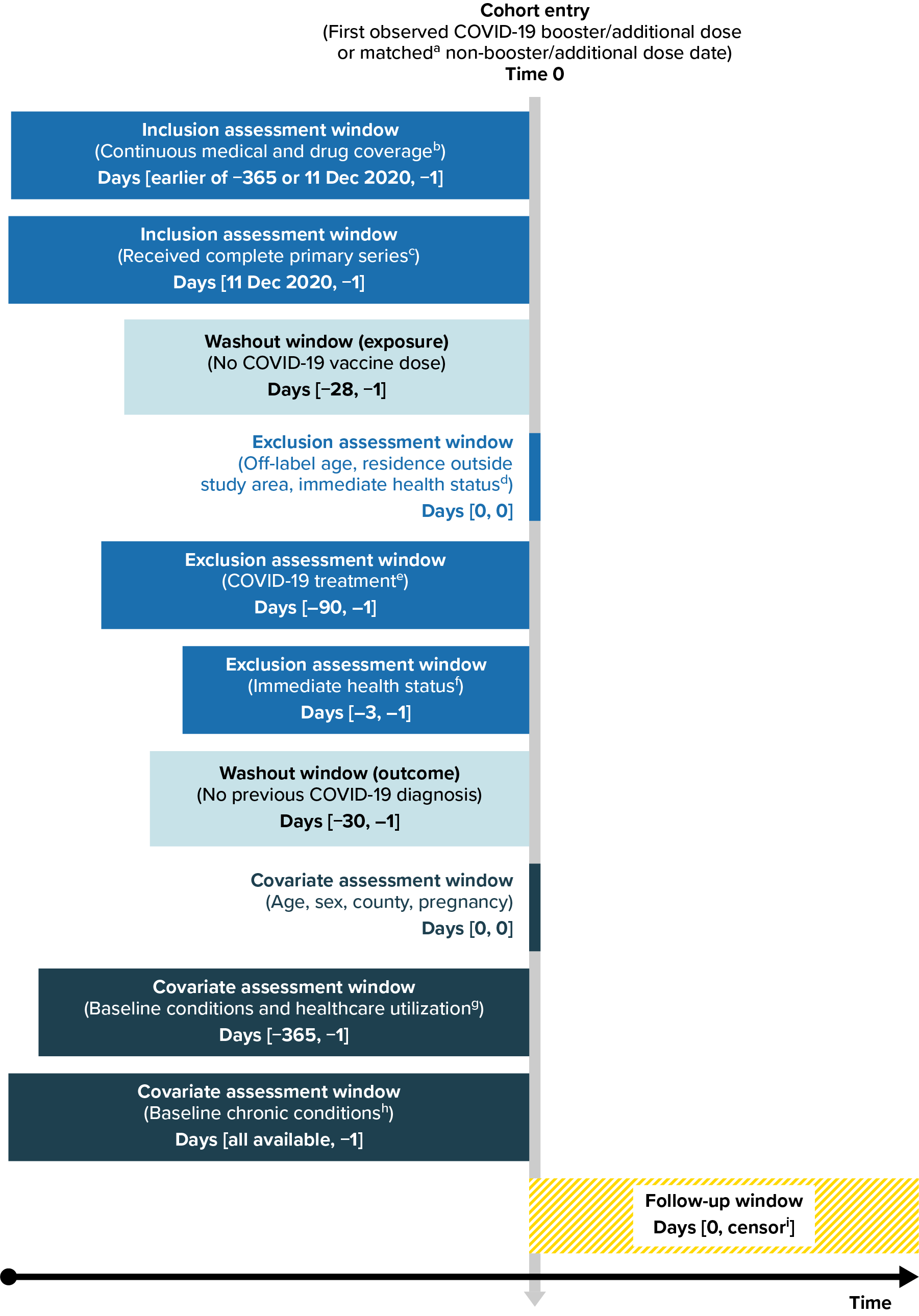


COVID‑19 = coronavirus disease 2019; ED = emergency department; LTC = long-term care.

^a^ Matching characteristics include calendar date of booster/additional dose receipt, age, sex, US county and state of residence, immunocompromised status, having a condition increasing risk of severe COVID-19, brand of primary series, and time since primary series.

^b^ Gaps in medical and pharmacy coverage < 32 days permitted.

^c^ Primary series is 1 dose of JNJ-7836735 or 2 doses of BNT162b2 or mRNA-1273 with the second dose occurring on or after 17 or 24 days, respectively, and within 42 days (inclusive) of the first dose, without any additional doses.

^d^ Hospitalization or LTC residence on Time 0.

^e^ COVID‑19 monoclonal antibodies or convalescent plasma.

^f^ Diagnoses of general acute symptoms (fever, nausea/vomiting, rash) and healthcare utilization (hospitalization, ED visit) serving as an indicator of health status at the time of vaccination.

^g^ Number of hospitalizations, number of emergency department visits, skilled nursing facility stay, influenza vaccination, pneumococcal vaccination, encounter for cancer screening, eye examination, colonoscopy, bone mineral density test, well-check/well-child preventive healthcare visit, arthritis, lipid abnormality, ambulance use/life support service, weakness, pregnancy completion before Time 0.

^h^ Autoimmune disorders, cancer, chronic kidney disease or renal disease, chronic liver disease, chronic lung diseases (e.g., asthma, chronic obstructive pulmonary disease [COPD], cystic fibrosis, pulmonary embolism), dementia or other neurological conditions, diabetes mellitus type 1 or 2, Down syndrome, heart conditions (e.g., heart failure, coronary artery disease, arrhythmias), hypertension, immunocompromised state, mental health conditions, obese or severely obese, sickle cell disease or thalassemia, stroke or cerebrovascular disease, tuberculosis, COVID-19 laboratory test performed (binary indicator of any test performed or none), COVID-19 diagnoses.

^i^ End of study period, end of continuous health plan enrollment, or receipt of any subsequent COVID-19 vaccine.

1. Attrition of the Matched Study Cohorts by Application of Eligibility Criteria and Matching Among Individuals Who Received a COVID-19 Booster/Additional Dose or Matched, Unboosted Comparators.

**A. Optum**


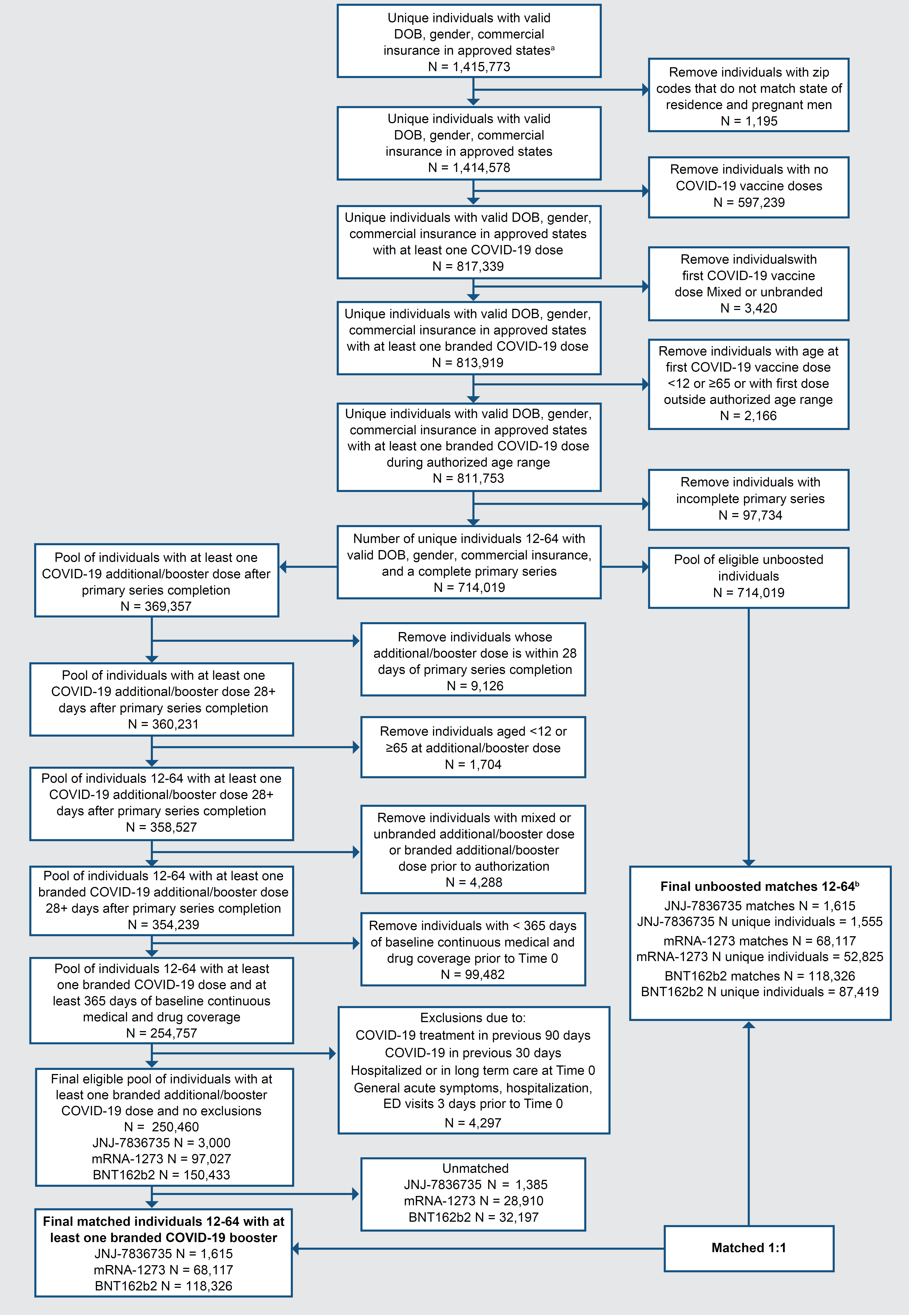


COVID-19 = coronavirus disease 2019; DOB = date of birth; ED = emergency department; IIS = immunization information system; US = United States.

^a^ 10 IIS jurisdictions from 10 unique US states.

^b^ Individuals in the unboosted group may also be included in the booster group with a different Time 0.

**B. CVS Health**


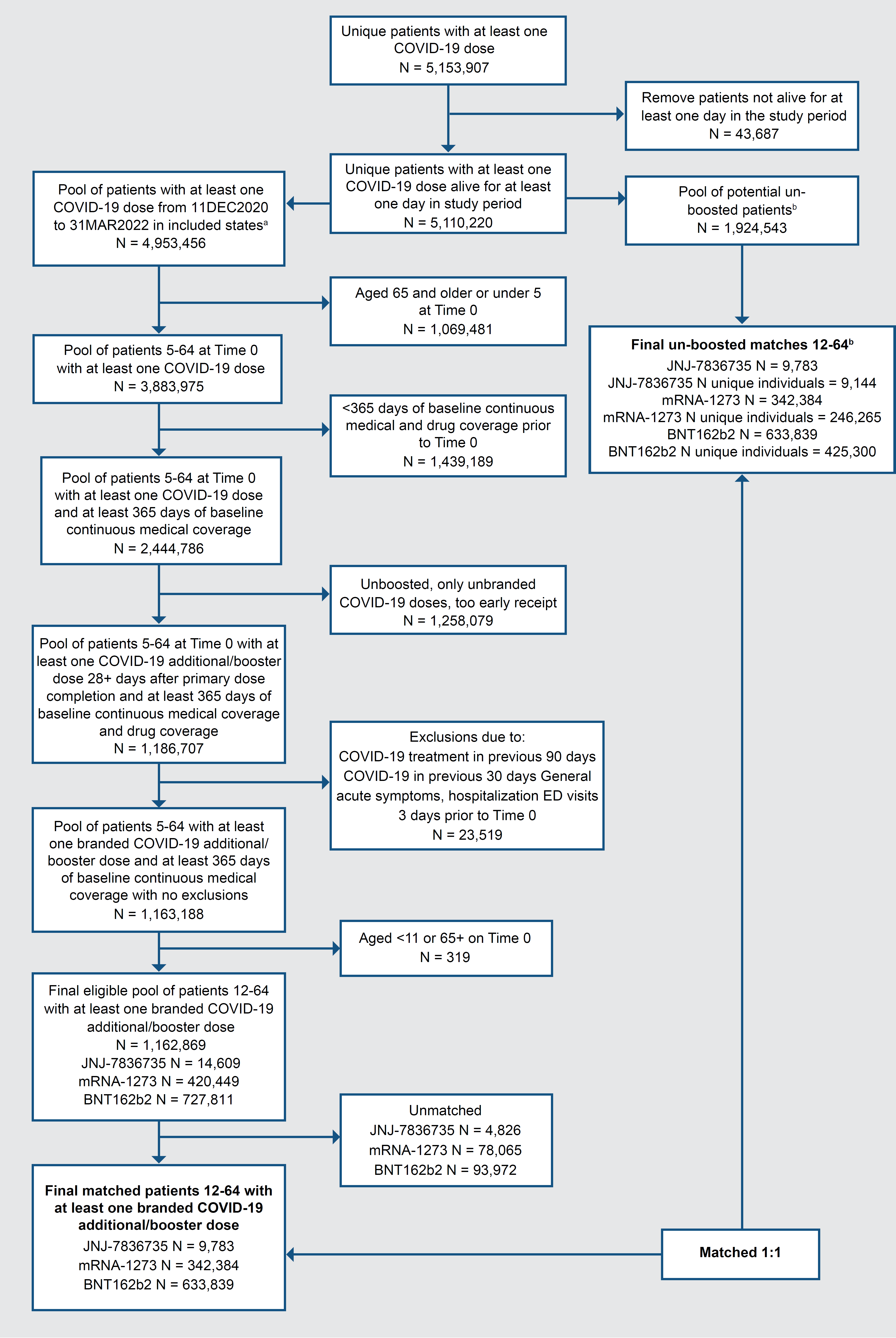


COVID-19 = coronavirus disease 2019; DOB = date of birth; ED = emergency department; IIS = immunization information system.

^a^ 11 IIS jurisdictions from 9 US states.

^b^ Individuals in the unboosted group may also be included in the boosted group with a different Time 0.

1. Distribution of Propensity Scores by Booster/Additional Dose Status for Each Vaccine-Specific Matched Cohort

A. Optum, BNT162b2


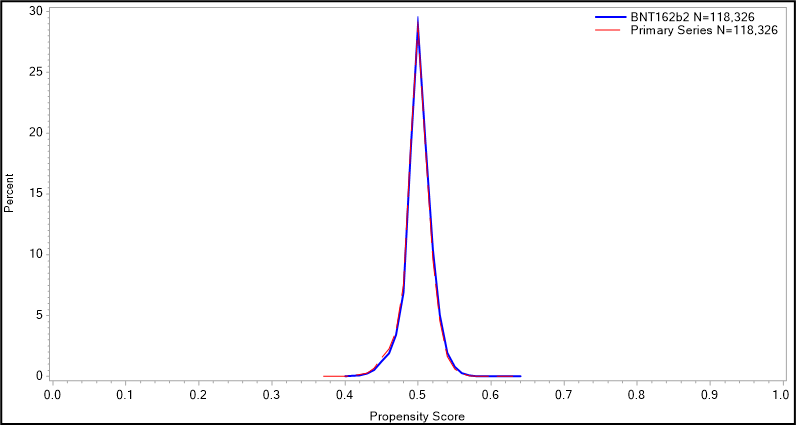


B. CVS Health, BNT162b2


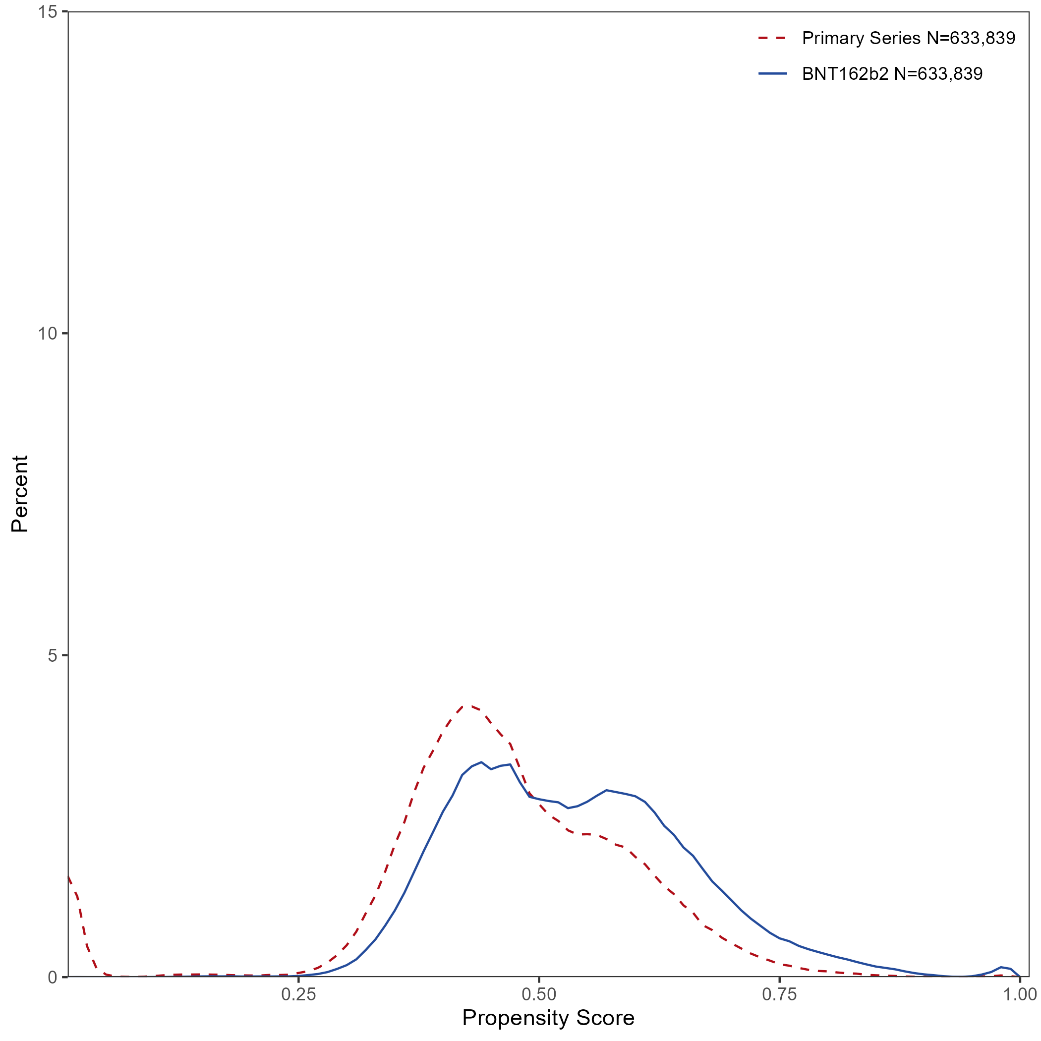


C. Optum, mRNA-1273

D. CVS Health, mRNA-1273


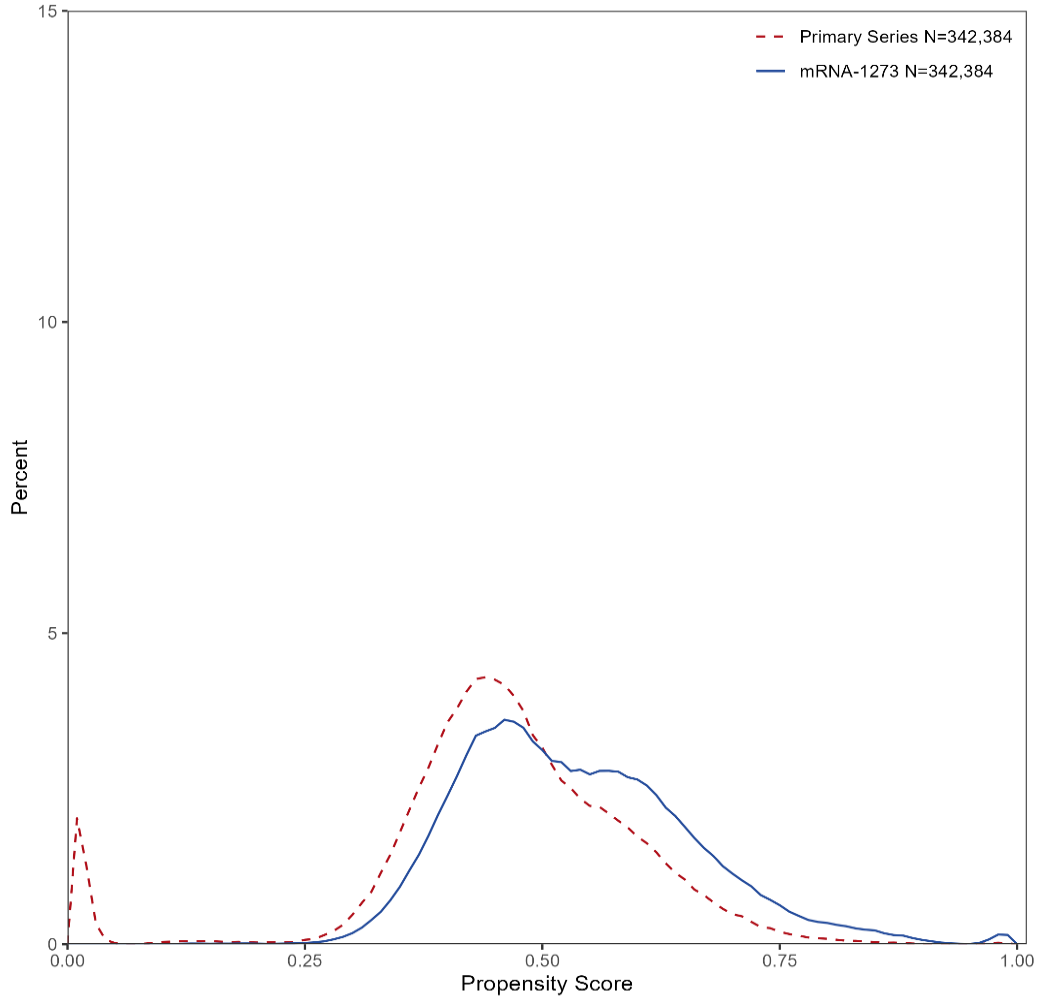


E. Optum, JNJ-7836735

F. CVS Health, JNJ-7836735


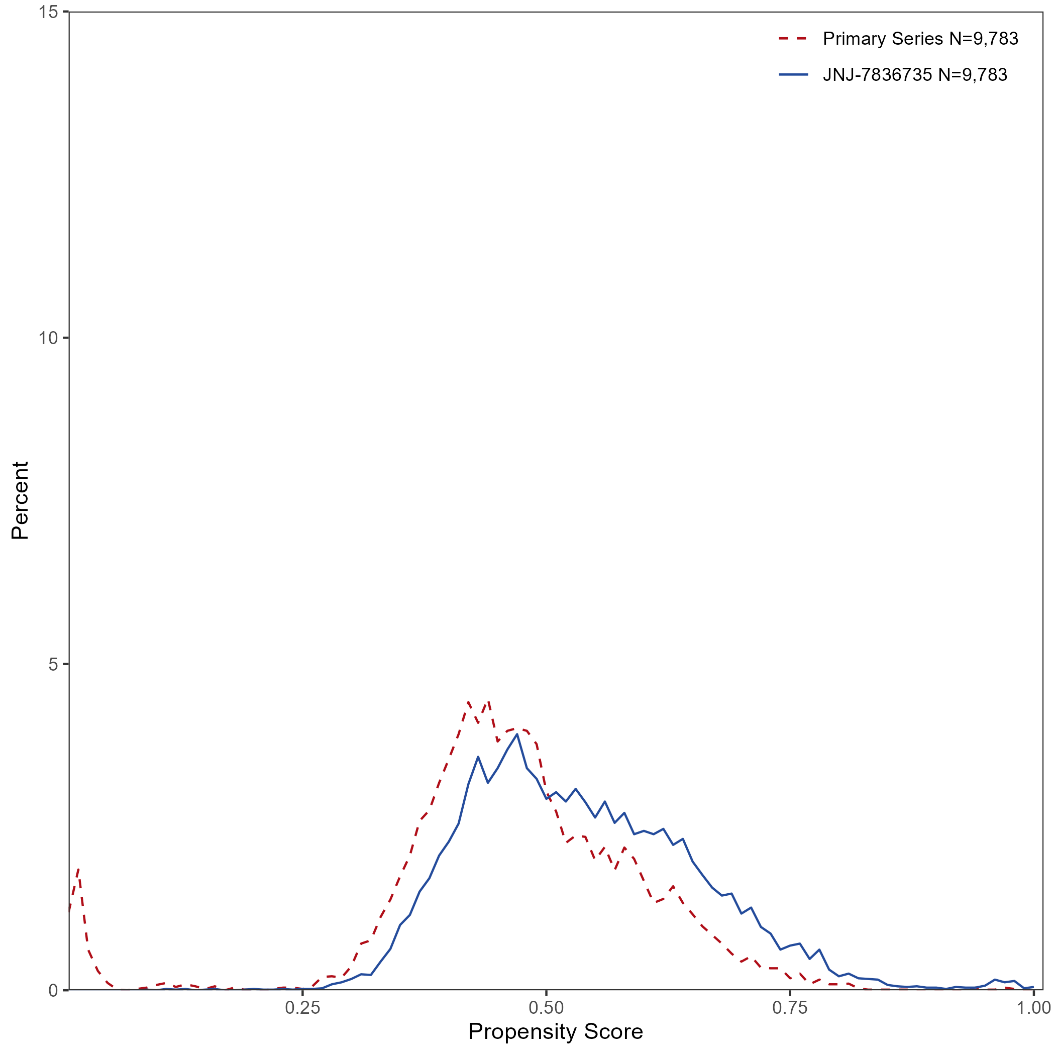


1. Inverse Probability of Treatment-Weighted Cumulative Incidence of COVID-19 Outcomes in Individuals Who Received a Booster/Additional Dose of COVID-19 Vaccine and Matched, Unboosted Comparators, by Vaccine Brand

A. BNT162b2: Medically Diagnosed COVID-19, Optum


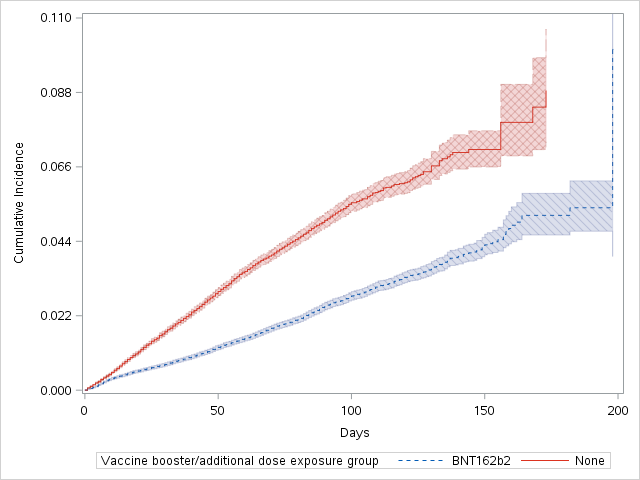


B. BNT162b2: Medically Diagnosed COVID-19, CVS Health


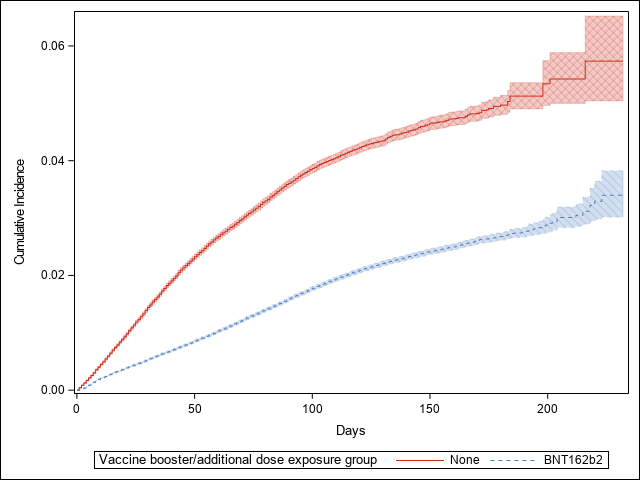


C. BNT162b2: Hospital/ED–diagnosed COVID-19, Optum


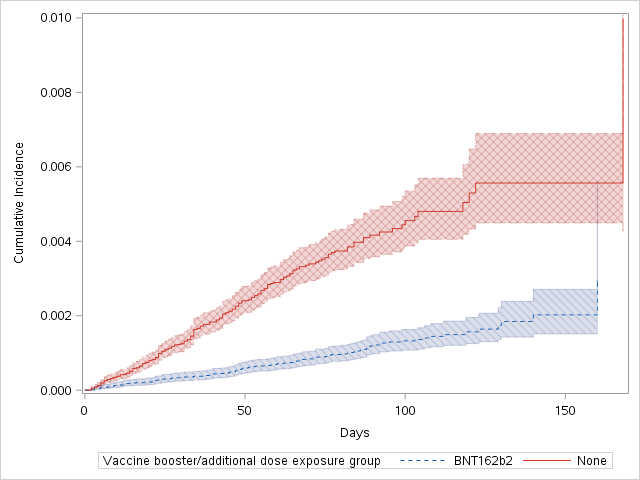


D. BNT162b2: Hospital/ED–diagnosed COVID-19, CVS Health


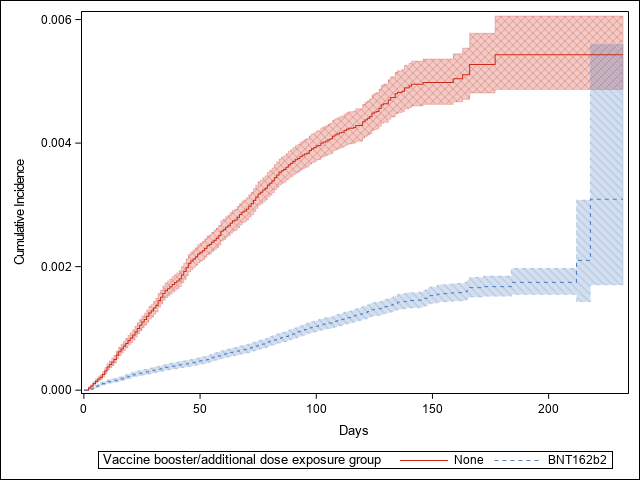


E. mRNA-1273: Medically Diagnosed COVID-19, Optum


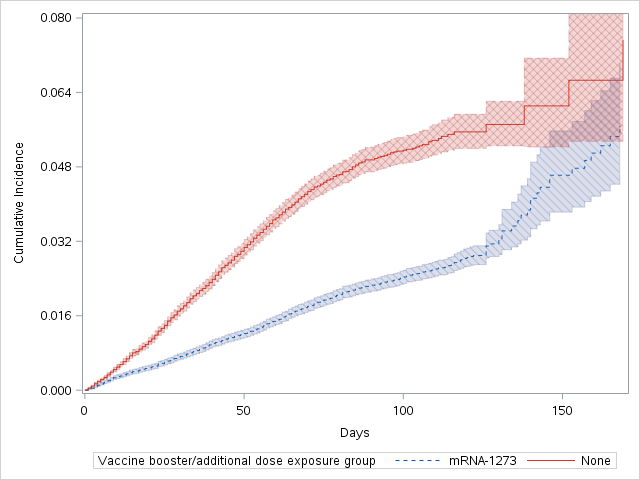


F. mRNA-1273: Medically Diagnosed COVID-19, CVS Health


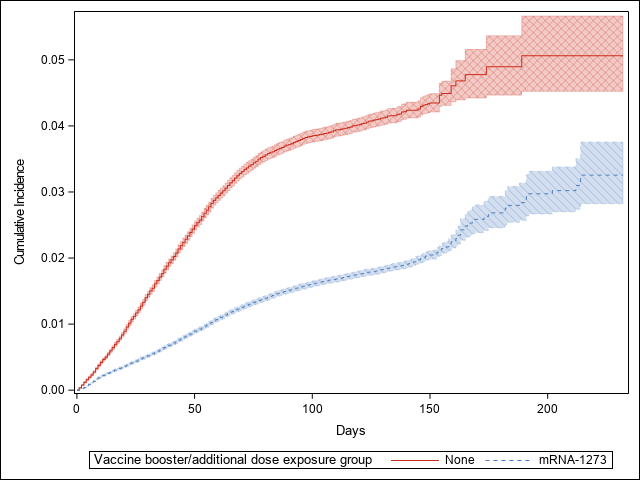


G. mRNA-1273: Hospital/ED–diagnosed COVID-19, Optum


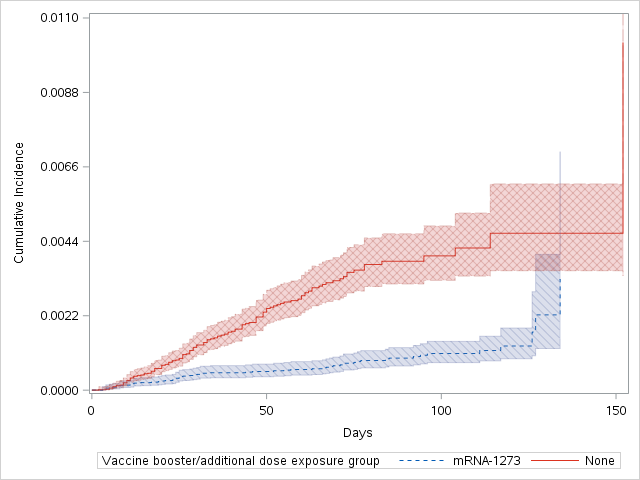


H. mRNA-1273: Hospital/ED–diagnosed COVID-19, CVS Health


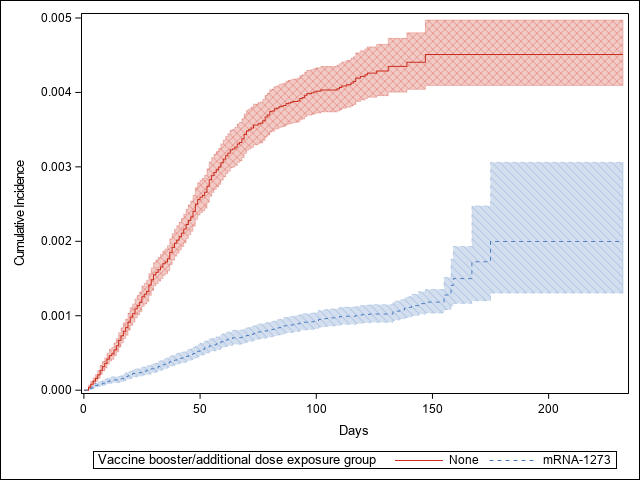


I. JNJ-7836735: Medically Diagnosed COVID-19, Optum


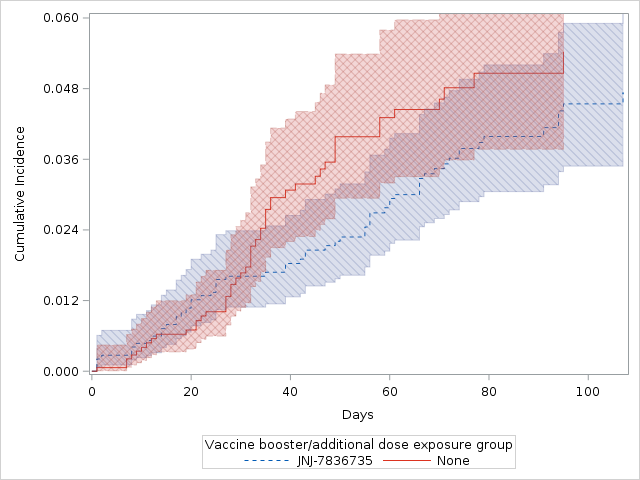


J. JNJ-7836735: Medically Diagnosed COVID-19, CVS Health


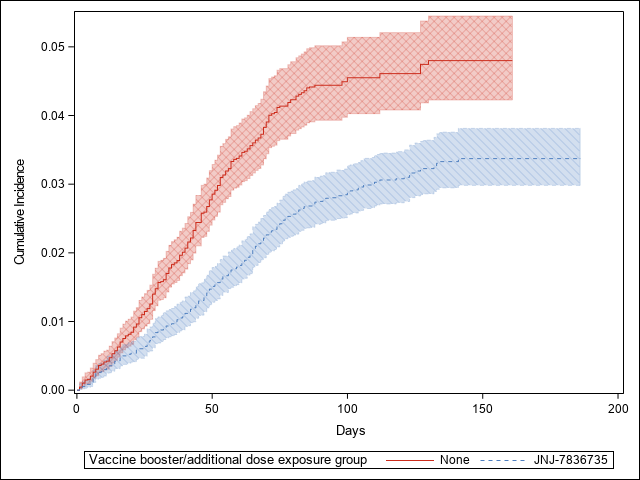


K. JNJ-7836735: Hospital/ED–diagnosed COVID-19, Optum


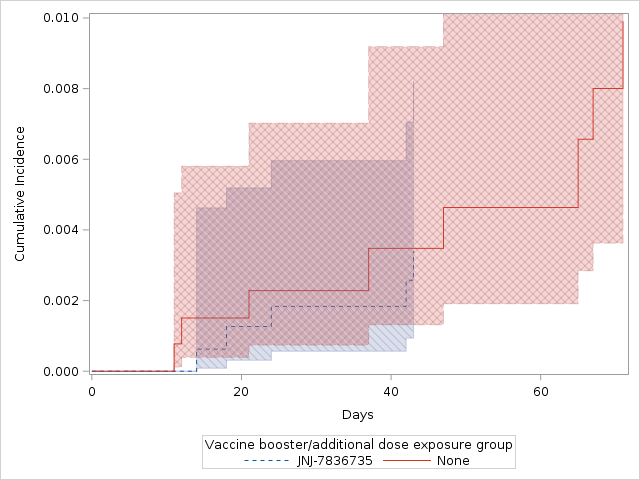


L. JNJ-7836735: Hospital/ED–diagnosed COVID-19, CVS Health


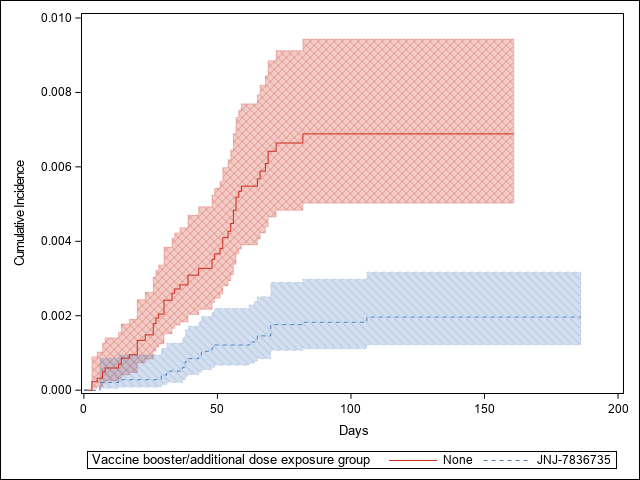


COVID-19 = coronavirus disease 2019; ED = emergency department.

1. Inverse Probability of Treatment-Weighted Cumulative Incidence of COVID‑19 Outcomes in Individuals Who Received a Booster/Additional Dose of COVID-19 Vaccine and Matched Unboosted Comparators, by Vaccine Brand, First 10 Days of Follow-up, Negative Control Outcome Analysis

A. BNT162b2, Medically Diagnosed COVID-19, Optum


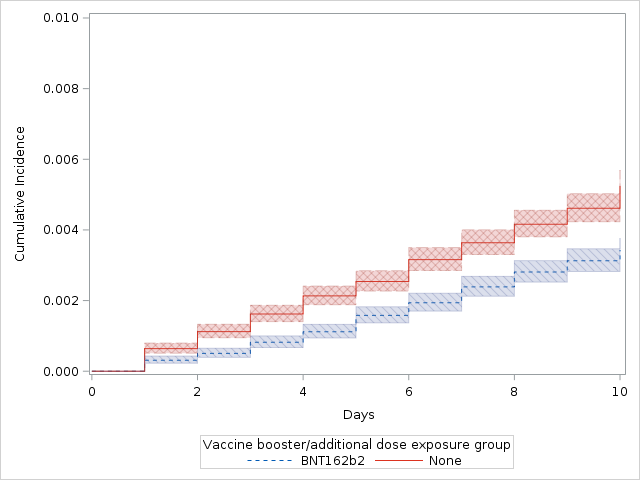


B. BNT162b2, Medically Diagnosed COVID-19, CVS Health


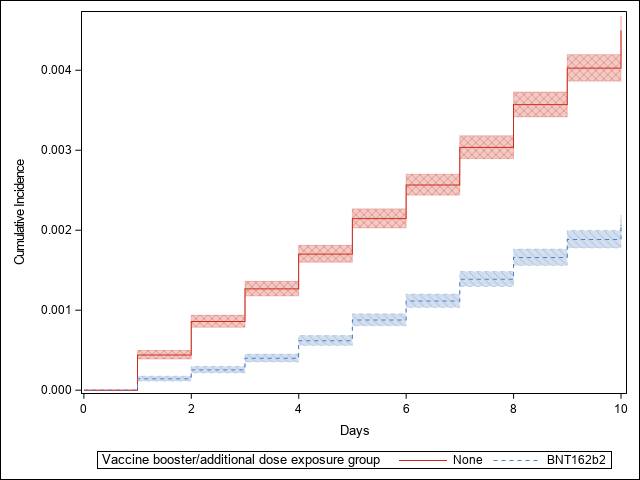


C. BNT162b2, Hospital/ED–diagnosed COVID-19, Optum


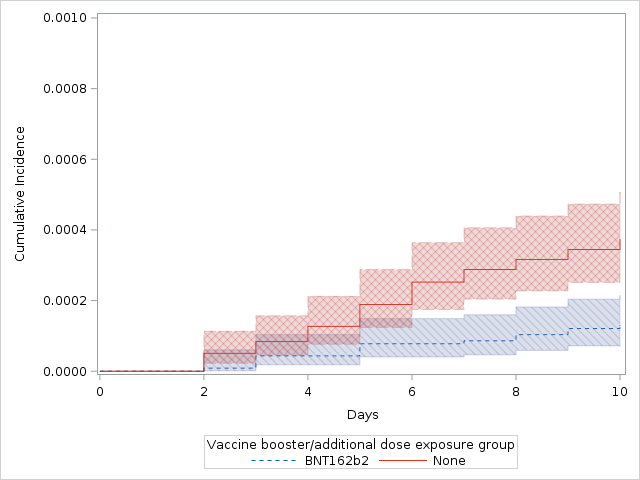


D. BNT162b2, Hospital/ED–diagnosed COVID-19, CVS Health


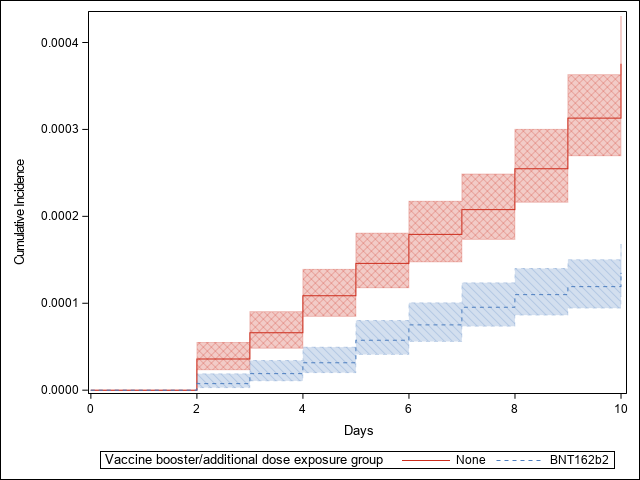


E. mRNA-1273, Medically Diagnosed COVID-19, Optum


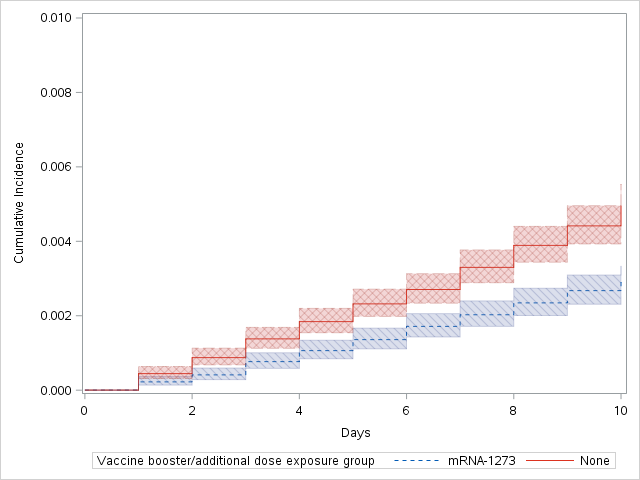


F. mRNA-1273, Medically Diagnosed COVID-19, CVS Health


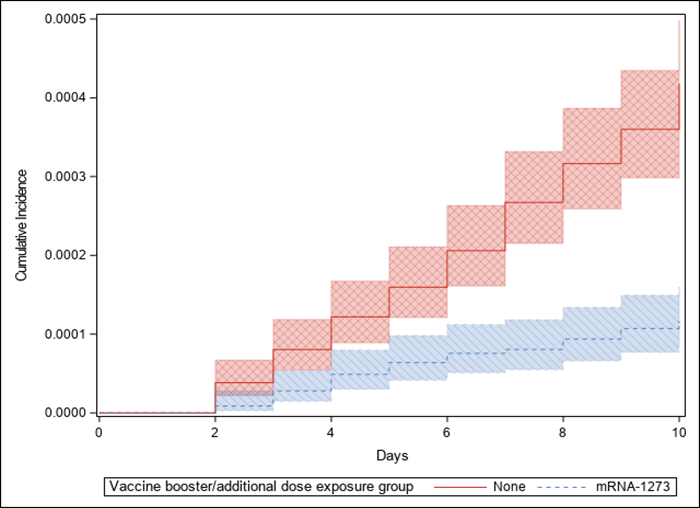


G. mRNA-1273, Hospital/ED–diagnosed COVID-19, Optum


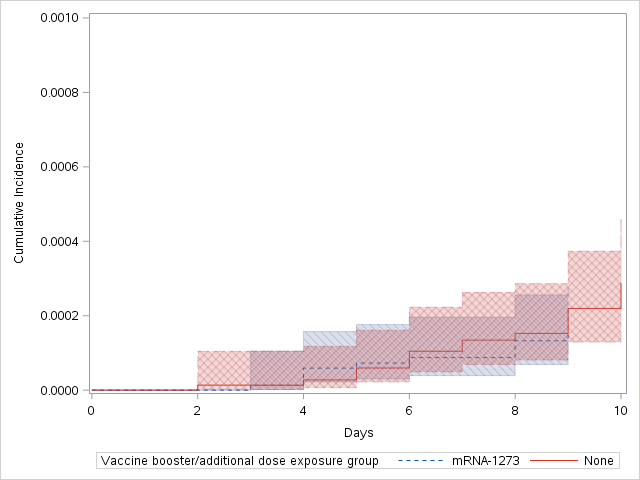


H. mRNA-1273, Hospital/ED–diagnosed COVID-19, CVS Health


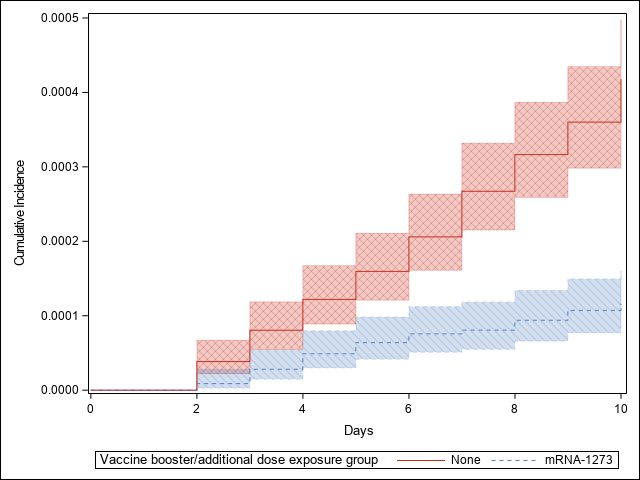


I. JNJ-7836735, Medically Diagnosed COVID-19, CVS Health


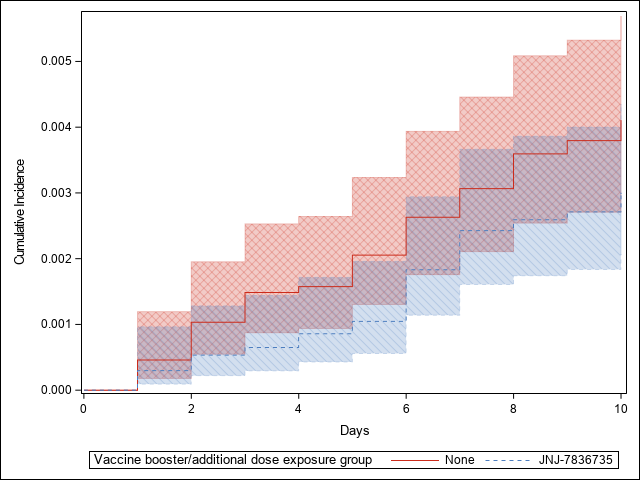


J. JNJ-7836735, Hospital/ED–diagnosed COVID-19, CVS Health


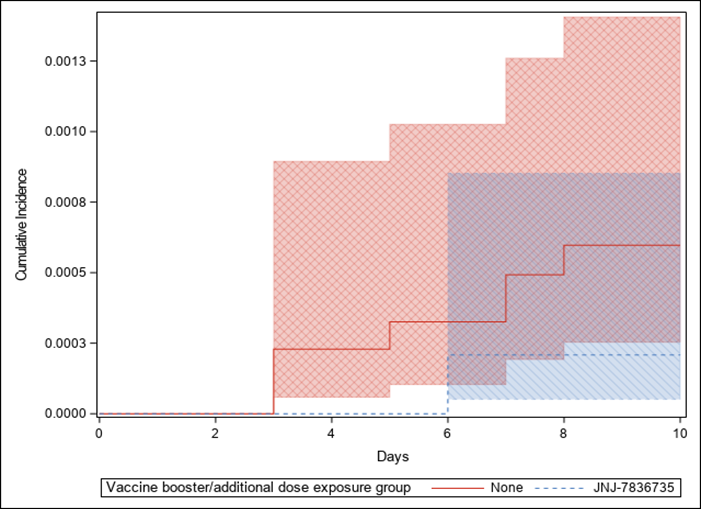


COVID-19 = coronavirus disease 2019; ED = emergency department.

Note: JNJ-7836735 analyses not performed in Optum due to small sample sizes.

# REFERENCES

1. Seeger J.D.; Schumock G.T.; Kong S.X. Estimating the rate of adverse drug reactions with capture-recapture analysis. *Am J Health Syst Pharm* **1996**, *53*, 178-81. doi:<http://dx.doi.org/10.1093/ajhp/53.2.178>.

2. FDA. Pfizer-BioNTech COVID-19 Vaccines. 2023. Available online: <https://www.fda.gov/vaccines-blood-biologics/coronavirus-covid-19-cber-regulated-biologics/pfizer-biontech-covid-19-vaccines> (accessed on 9 August 2023).

3. FDA. Moderna COVID-19 Vaccines. 2023. Available online: <https://www.fda.gov/vaccines-blood-biologics/coronavirus-covid-19-cber-regulated-biologics/moderna-covid-19-vaccines> (accessed on 9 August 2023).

4. FDA. Janssen COVID-19 vaccine. 2022. Available online: <https://www.fda.gov/emergency-preparedness-and-response/coronavirus-disease-2019-covid-19/janssen-covid-19-vaccine> (accessed on 2 March 2022).
